# Nickel(II) Complexes Derived from Schiff Base Ligands Designed as Electrode Materials in Asymmetric Supercapacitor Coin Cells for Enhanced Energy Storage Performance

**DOI:** 10.1021/acs.langmuir.5c04823

**Published:** 2026-01-12

**Authors:** Ibrahim Waziri, Tunde L. Yusuf, Alfred J. Muller, Charity N. Mbileni Morema, Kaushik Mallick, Sarit K. Ghosh

**Affiliations:** † Department of Chemical Sciences, 61799 University of Johannesburg, P.O. Box 524, Auckland Park, Johannesburg 2006, South Africa; ‡ Department of Chemistry, Faculty of Natural and Agricultural Sciences, 56410 University of Pretoria, Private Bag X20, Hatfield, Pretoria 0028, South Africa; § NM Envirotech Solutions, Midlands Estate, Centurion 1692, South Africa

## Abstract

In this study, three nickel­(II)-based Schiff base complexes, derived from the condensation of 2-hydroxybenzaldehyde with 2-bromo-4-chloroaniline (C1), 2-bromo-4-methylaniline (C2), and 2-iodo-4-nitroaniline (C3), were synthesized using a one-pot in situ reaction strategy without isolating the corresponding ligands. The complexes were characterized using standard spectroscopic techniques, and the solid-state structures for C1 and C2 were determined by a single-crystal X-ray diffraction analysis. The Schiff base-derived complexes (C1, C2, and C3) were fabricated as an electrode material, and their electrochemical performance was evaluated in a 2 M KOH aqueous electrolyte. Cyclic voltammetry confirmed their pseudocapacitive behavior, as evidenced by distinct redox peaks. Among the three electrodes, the 2-iodo-4-nitroaniline-based complex (C3) exhibited a superior charge-storage capability and higher dielectric polarizability. At 1 A·g^–1^, the C3 electrode delivered a maximum specific capacitance of ∼330 F·g^–1^ and retained ∼92.5% of its capacitance after 10,000 charge–discharge cycles at 5 A·g^–1^. An asymmetric (AC//C3) supercapacitor coin cell operating at 1.6 V delivered a specific capacity of ∼98.3 C·g^–1^ (61.43 F·g^–1^ or ∼27.3 mAh·g^–1^) at 0.5 A·g^–1^. The device achieved an energy density of ∼21.8 Wh·kg^–1^ with a power density of ∼378.3 W·kg^–1^ at 0.5 A·g^–1^, reaching a maximum power density of ∼1089 W·kg^–1^ at 4.0 A·g^–1^. Furthermore, two coin cells connected in series produced ∼2.91 V, sufficient to power a red LED, demonstrating the practical applicability of the C3-based electrode system for real-world energy storage devices.

## Graphical abstract

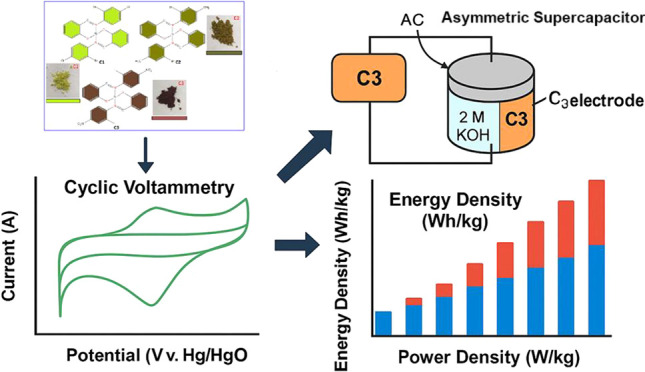

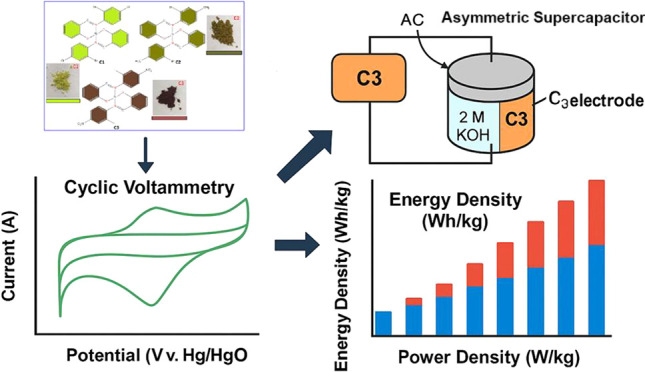

## Introduction

In recent years, the global demand for high-efficiency, durable, and sustainable energy storage systems has increased significantly due to the rapid development of renewable energy technologies and the electrification of transportation.^1–4^ Supercapacitors have emerged as promising candidates for next-generation energy storage owing to their exceptional power density, rapid charge–discharge capability, and long cycle life compared to conventional batteries.^5–10^ These advantages enable their use in portable electronics, hybrid electric vehicles, and grid-based storage systems.^11–14^ However, the relatively low energy density of supercapacitors remains a persistent limitation, necessitating the development of advanced electrode materials with improved electrochemical performance.^5,15–17^ To address this challenge, diverse categories of electrode materials, including activated carbon, graphene, and carbon nanotubes, have been studied extensively.^18–22^ Although these carbon-based materials exhibit good conductivity and high surface area, their limited pseudocapacitive character constrains their achievable specific capacitance.^23–25^ Transition-metal-based materials (oxides, hydroxides, sulfides) provide higher charge-storage capability via reversible redox processes, yet they often suffer from poor conductivity, structural instability, and multistep synthesis routes, restricting their practical applicability.^26–29^

In this context, organometallic systems, particularly those derived from Schiff base ligands, have received increasing attention as promising pseudocapacitive materials.^30–33^ Schiff bases, formed through the condensation of primary amines with carbonyl compounds, possess excellent tunability and strong binding affinity toward metal ions, enabling the formation of structurally robust complexes with diverse redox properties.^34–36^ Nickel complexes, in particular, have demonstrated attractive electrochemical features such as high theoretical capacitance, narrow redox potentials, and environmental compatibility.^30,37–41^ Several studies have reported Schiff-base-derived materials with encouraging electrochemical performance, including Ni–OTTP frameworks,^31^ Salphen-type nickel Schiff base polymers,^42^ nitrogen-rich carbon spheres from Schiff base precursors,^43,44^ and cobalt- or nickel-embedded polymeric or MOF-based systems.^45–48^ These works collectively demonstrate the versatility of Schiff base chemistry in energy storage applications. Despite this progress, a critical knowledge gap remains: the role of systematic and targeted ligand electronic modification on the dielectric response, redox behavior, and overall supercapacitor performance of discrete molecular Ni­(II)–Schiff base complexes has not been fully elucidated. Most reported Schiff base-based supercapacitor systems rely on polymeric, carbonized, or MOF-derived architectures, whereas studies involving well-defined, mononuclear Ni­(II) complexes constructed through controlled substituent tuning (electron-donating vs halogen electron-withdrawing groups) remain scarce. In addition, asymmetric devices employing such molecular Ni complexes in practical coin cell configurations are underexplored, particularly those linking molecular electronic structures to macroscopic device behavior.^49^ Recent progress in MOFs, perovskites, and nanocomposites demonstrates that introducing conductive scaffolds (CNTs, MXenes) enhances electron transport and cycling stability.^50–52^ However, these hybrid systems do not isolate molecular-level structure–property relationships or allow direct interrogation of how ligand substituents modulate dielectric polarizability, band gap, redox kinetics, and long-term cycling behavior—relationships critical for rational material design.

To address these gaps, the present study reports a one-pot in situ synthetic strategy for three discrete Ni­(II)–Schiff base complexes (C1–C3), derived from 2-hydroxybenzaldehyde and substituted anilines containing electron-donating (methyl) and various electron-withdrawing halogen (Cl, Br, I) and nitro groups. This rational ligand modification enables probing of how substituent electronics influence charge-transfer capabilities, dielectric properties, and pseudocapacitive behavior. The complexes were evaluated as electrode materials in a 2 M KOH electrolyte, revealing distinct correlations between ligand electronic effects and electrochemical properties.^53,54^ Among the synthesized complexes, the 2-iodo-4-nitroaniline-based complex (C3) exhibited superior electrochemical activity, high cycling stability, and improved dielectric polarizability. An asymmetric coin cell (AC//C3) was subsequently fabricated, representing a rare example of a molecular Ni–Schiff base-derived asymmetric supercapacitor device. After potential optimization, two such coin cells connected in series successfully powered a red LED, demonstrating real-world applicability.

Thus, the novelty of this work lies in (i) the systematic electronic modulation of discrete Ni–Schiff base complexes; (ii) the establishment of a mechanistic structure–property–performance correlation involving dielectric behavior, optical band gap, and redox kinetics; and (iii) the construction and validation of an asymmetric coin cell device based on a mononuclear Schiff base–nickel complex, an approach not yet widely reported. This study therefore provides both new molecular insights and a practical device-level demonstration, advancing the fundamental and applied potential of Schiff base–metal complexes for next-generation supercapacitor technologies.

## Experimental Section

### Chemicals and Reagents

For this study, we utilized analytical-grade chemicals and reagents obtained from Merck Pty Ltd. These materials were used without additional purification. The chemicals employed in this research included 2-bromo-4-chloroanilline, 2-bromo-4-methylanilline, 2-iodo-4-nitroanilline, 2-hydroxybenzaldehyde, nickel chloride hexahydrate, methanol, dichloromethane, hexane, and formic acid.

### General Procedure for the Synthesis of the Complexes

Nickel­(II) complexes C1–C3 were synthesized using a one-pot in situ method without isolating the ligands. The synthesis procedures for the complexes were adapted from previously reported literature.^55,56^ The specific details for the synthesis of each complex are as follows: A methanolic solution (1.0 mmol) of the primary amines: 2-bromo-4-chloroaniline, 2-bromo-4-methylaniline, or 2-iodo-4-nitroaniline was added to a stirring methanolic solution (1.0 mmol) of 2-hydroxybenzaldehyde at room temperature, followed by the addition of three drops of formic acid. This addition resulted in the formation of various colors in each mixture, which were stirred for 1 h. Subsequently, a solution of nickel chloride hexahydrate (119 mg, 0.5 mmol) in 10 mL of methanol was added to each solution and further stirred for 3 h. This addition led to the formation of a green powder in all reactions, which was then filtered, washed with methanol under vacuum, and allowed to dry, yielding complexes C1–C3. To grow crystals of each complex, vapor diffusion of hexane into a concentrated solution of dichloromethane (DCM) was used, resulting in the formation of dark blue crystals for the C1 and C2 complexes. The reaction pathway is illustrated in Scheme 1.

**Scheme 1:**
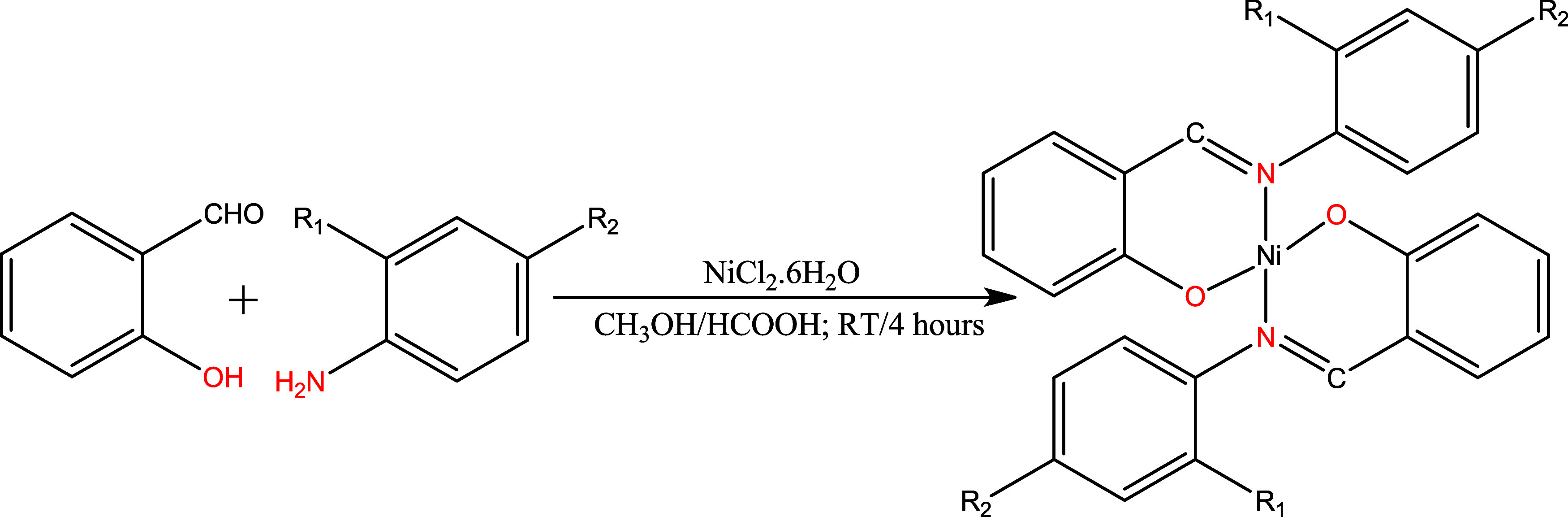
Synthetic Route for the Formation of the Ni­(II)–Schiff Base Complexes C1–C3^a^

a Substituent patterns: **C1** (*R*_1_ = Br, *R*_2_ Cl), **C2** (*R*_1_ = Br, *R*_2_ = CH_3_), and **C3** (*R*_1_ = I, *R*_2_ = NO_2_).

#### Bis­(*E*)-2-(((2-bromo-4-chlorophenyl)­imino)­methyl)­phenolatenickel­(II) (C1)

A green solid, yield: 71% (202.2 mg), mp: 226 °C; ^1^H NMR (500 MHz, DMSO-*d*_*6*_); δ­(ppm): 5.92 (d, 1H, 8.5 Hz, Ar–H), 6.53 (t, 1H, *J* = 7.5 Hz, Ar–H), 7.23 (t, 1H, *J* = 7.5 Hz, Ar–H), 7.43 (d, 1H, *J* = 9.0 Hz, Ar–H), 7.49 (d, 1H, *J* = 8.0 Hz, Ar–H), 7.57 (d, 1H, *J* = 6.5 Hz, Ar–H), 7.88 (s, 1H, Ar–H,), 8.08 (s, 1H, HC=N); ^13^C NMR (125 MHz, DMSO-*d*_*6*_); δ­(ppm): 114.8, 119.5, 119.6, 119.7, 120.0, 127.6, 127.8, 130.8, 131.1, 135.4, 136.3 (Ar–C), 164.4 (C–O, Ar–C), 164.7 (HC=N); FTIR_ATR_; λ­(cm^–1^): 1598 (C=N), 1432 (C–N), 746 (C–Cl), 557 (Ni–N), 443 (Ni–O); UV–vis: (DMSO, 10^–3^ M); υ (nm): 250 (π→π*), 300 (n→π*), 435 (d→d); Elemental Analysis (CHN) for C_26_H_16_Br_2_Cl_2_N_2_NiO_2_; calcd: C, 46.07, H, 2.38, N, 4.13, Ni, 8.66; found: 46.03, H, 2.38, N, 4.10, Ni, 8.64; molecular weight for C_26_H_16_Br_2_Cl_2_N_2_NiO_2_; calcd: 673.8309 [M]^+^; found = 673.4890 [M]^+^.

#### Bis­(2-{[(2-bromo-4-methylphenyl)­imino]­methyl}­phenolato)-nickel­(II) (C2)

A dark green solid, yield: 50% (158.5 mg), mp: 195 °C; ^1^H NMR (500 MHz, DMSO-*d*_*6*_); δ­(ppm): 2.38 (s, 3H, CH_3_), 5.90 (d, 1H, *J* = 8.5 Hz, Ar–H), 6.49 (t, 1H, *J* = 7.0 Hz, Ar–H), 7.17 (t, 1H, *J* = 7.5 Hz, Ar–H), 7.27 (d, 2H, *J* = 7.5 Hz, Ar–H), 7.41 (d, 1H, *J* = 7.5 Hz, Ar–H), 7.55 (s, 1H, Ar–H), 8.00 (s, 1H, HC=N); ^13^C NMR (125 MHz, DMSO-*d*_*6*_); δ­(ppm): 20.1 (CH_3_), 114.4, 119.6, 119.7, 128.1, 131.8, 135.2, 137.5, 144.8, (Ar–C), 164.4 (C–O, Ar–C), 164.5 (C=N); FTIR_ATR_; λ­(cm^–1^): 1600 (C=N), 1428 (C–N), 760 (C–Br), 557 (Ni–N), 471 (Ni–O); UV–vis: (DMSO, 10^–3^ M); υ (nm): 274 (π→π*), 342 (n→π*), 442 (d→d); Elemental Analysis (CHN) for C_28_H_22_Br_2_N_2_NiO_2_; calcd: C, 52.80, H, 3.48, N, 4.40, Ni, 9.21; found: C, 52.76, H, 3.46, N, 4.38, Ni, 9.19; molecular weight for C_28_H_22_Br_2_N_2_NiO_2_; calcd: 633.9402 [M]^+^; found = 633.1966 [M]^+^.

#### Bis­(*E*)-2-(((2-iodo-4-nitrophenyl)­imino)­methyl)­phenolatenickel­(II) (C3)

A brown solid, yield: 56% (222.0 mg), mp: 262 °C; ^1^H NMR (500 MHz, DMSO-*d*_*6*_); δ­(ppm): 6.11 (d, 1H, *J* = 8.5 Hz, Ar–H), 6.76 (d, 1H, *J* = 7.5 Hz, Ar–H), 6.77 (d, 1H, *J* = 6.5 Hz, Ar–H), 6.99 (d, 1H, *J* = 8.5 Hz, Ar–H), 7.36 (t, 1H, *J* = 8.0 Hz, Ar–H), 7.38 (d, 1H, *J* = 6.5 Hz, Ar–H), 7.48 (d, 1H, *J* = 6.5 Hz, Ar–H), 7.66 (d, 1H, *J* = 7.5 Hz, Ar–H), 8.55 (s, 1H, HC=N); ^13^C NMR (125 MHz, DMSO-*d*_*6*_); δ­(ppm): 116.0, 118.9, 120.9, 122.6, 125.7, 128.1, 131.3, 132.7, 134.5 (Ar–C), 160.5 (C–O, Ar–H), 188.9 (C=N); FTIR_ATR_; λ­(cm^–1^): 2900 (C–H), 1595 (C=N), 1438 (C–N), 1320 (NO_2_), 754 (C–I), 535 (Ni–N), 468 (Ni–O); UV–vis: (DMSO, 10^–3^ M); υ (nm): 232 (π→π*), 290 (n→π*), 530 (d→d); Elemental Analysis (CHN) for C_26_H_16_I_2_N_4_NiO_6_; calcd: C, 39.38, H, 2.03, N, 7.07, Ni, 7.40; found: C, 39.36, H, 2.01, N, 7.05, Ni, 7.39; molecular weight for C_26_H_i6_I_2_N_4_NiO_6_; calcd: 792.8591 [M]^+^; found = 793.1534 [M]^+^.

### Measurements and Instrumentations

The elemental compositions (carbon, hydrogen, and nitrogen) of these compounds were determined by using a VarioElementar III microbe CHNS analyzer. Infrared spectra were recorded using a Tensor 27 Bruker and a PerkinElmer FTIR spectrometer BX, covering the range of 4000–400 cm^–1^. Electronic absorption spectra were obtained using a Shimadzu UV–vis 1800 spectrophotometer in DMSO at room temperature, spanning the range of 800–200 nm. The ^1^H and ^13^C NMR spectra were acquired on Bruker 500 and 125 MHz spectrometers, respectively, at room temperature. Chemical shifts are reported in parts per million (ppm), relative to tetramethylsilane as the internal standard for both ^1^H and ^13^C NMR. Mass spectra were acquired by using a Waters Acquity UPLC Synapt G2 HD mass spectrometer. X-ray photoemission spectroscopy (XPS, VG Multilab 2000) was performed on pristine (fabricated on Ni-form) and post-cycle C3 systems to determine elemental composition and oxidation states. The post-cycle electrode was rinsed with deionized water and then ethanol to remove the electrolyte (KOH) and finally dried out under an inert atmosphere for XPS analysis. All electrochemical measurements such as cyclic voltammetry (*C–V*), galvanostatic charge–discharge (GCD) process, material stability (cycles), and electrochemical impedance spectra (EIS) were carried out using a Bio-Logic SP-300 potentiostat. During the measurements, 90% *iR* compensation was applied via the EC-Lab software control program to minimize the ohmic drop. The SP-300 potentiostat adjusts the applied potential in real time to counteract the *iR* drop. ESR is obtained from the x-intercept of the Nyquist plot (EIS spectra) in the high-frequency region. The dielectric properties of the fabricated thin-film C1, C2, and C3 materials were investigated by using an LCR meter (HIOKI-3536) interface with a computer-controlled probe chamber under a frequency sweep from 200 Hz to 1 MHz at ambient temperature (25 °C).

### Single X-ray Preparation

Dark blue crystals suitable for single-crystal XRD were grown by vapor diffusion for C1 and C2. About 5 mg of compounds was dissolved in 2 mL of dichloromethane and layered with hexane, which afforded single crystals suitable for data collection for C1 and C2. The crystalline molecules show two of the ligands chelating the nickel­(II). The details for each of the crystals are given in the Crystallographic Analysis section.

### Electrochemical Analysis

#### Fabrication of C1, C2, and C3 Electrodes

The working electrodes of C1, C2, and C3 materials were prepared by thoroughly mixing 80 wt % active material (C1, C2, and C3), 10 wt % conducting carbon black (CB), and 10 wt % poly­(vinylidene fluoride) (PVDF) as a binder for 2 h in the presence of *N*-methyl-2-pyrrolidone (NMP) solvent. Before electrode preparation, a piece of Ni-foam (standard thickness ∼1.0 mm, ∼95% porosity, average pore size ∼450 μm) was initially treated with a mild acid (0.1 M HCL solution) to degrease thoroughly. Finally, the Ni-foam was ultrasonicated for 40 min in an acetone/ethanol solution, rinsed with deionized water, and dried at 60 °C. The obtained homogeneous slurry was deposited (drop casting) on the Ni-foam (active surface area ∼1 × 1 cm^2^) and then vacuum-dried at 60 °C for 12 h to remove the residual NMP. The dry electrode was pressed through a roll press at 50 MPa to improve the contact and porosity of the Ni-foam without damaging the surface. The mass loading of the working electrodes was approximately 2.2, 2.4, and 2.3 mg cm^–2^ for C1, C2, and C3, respectively.. A platinum (Pt) wire and a saturated calomel electrode (SCE) were used as the counter electrode and reference electrode, respectively. The electrochemical performances of the fabricated electrodes are investigated in a 2 M KOH electrolyte within the potential window from 0 to 0.6 V.

#### Fabrication of an AC//C3 Asymmetric Supercapacitor Coin Cell

An asymmetric supercapacitor AC//C3 device was fabricated on the Ni-foam as a coin cell (LIR2032, 20 mm diameter, 3.2 mm thickness) format. The cell consists of activated carbon (AC) as a negative electrode, C3 as a positive electrode, and a 2 M KOH aqueous electrolyte. The coin cell is assembled inside an Ar-filled glovebox (<10 ppm level, chamber temperature ∼25 °C). The coin cell consists of a metallic case (top/bottom sides), a stainless steel spacer (top/bottom sides) of coin cell diameter (thickness ∼0.5 mm), a separator (thickness ∼200 μm), a spring coil (stainless steel) that sits under the spacer, and a sealing gasket (O-ring, polymer insulator type), which is placed inside the coin cell to provide compression when crimped. The electrolyte consisted of a 2 M KOH aqueous solution, with an electrolyte volume of ∼100 μL per cell to ensure full wetting of both electrodes and the separator.

A microporous glass fiber separator (Whatman filter paper, thickness ∼200 μm) was employed and presoaked in the same electrolyte for at least 12 h prior to assembly to achieve complete electrolyte penetration and minimize internal resistance. The filter was placed between the negative and positive electrodes, acting as a separator inside the coin cell. The assembled stack crimps inside the glovebox and applies a steady pressure until it tightens into a coin cell. Cells were crimped under moderate pressure and operated at ≤1.6 V to avoid gas evolution. No leakage or swelling was observed after extended cycling. The cell maintained the charge balance (*q*+ = *q*−) between the positive and negative electrodes. The equivalent mass ratio was calculated from the balance relation *m*_+_/*m*_–_ = *C*_p–_Δ*V*_–_/*C*_p+_Δ*V*_+_, where *m*_+_, *C*_p+_, and Δ*V*_+_ and *m*_–_, *C*_p–_, and Δ*V*_–_ are the mass, specific capacitance, and potential window of positive and negative electrodes, respectively. The optimum mass ratio (*m*_+_/*m*_–_) of ∼0.40 is calculated from the cyclic voltammetry performances of the AC and C3 electrodes. The device was fabricated by using a positive electrode (cathode) active mass *m*_+_ of 1.6 mg, and a negative electrode (anode) active mass *m*_–_ of 4.0 mg, with a total mass of ∼5.6 mg. The active materials were coated on a circular nickel foam disk with ∼11.28 mm diameter and a geometric area *A* of ∼1.0 cm^2^. This corresponds to areal mass loadings of ∼1.6 mg·cm^–2^ (positive) and ∼4.0 mg·cm^–2^ (negative), within the tolerances of ±5%. Active masses were measured on an analytical microbalance (readability 0.01 mg) after drying at 60 °C for 12 h. Reported electrochemical metrics were obtained from at least three independent cells and are given as mean ± standard deviation.

#### Fabrication of Thin-Film Devices (C1, C2, and C3) for Dielectric Properties

Thin-film C1, C2, and C3 devices were fabricated according to the following protocol. Initially, the slurry was prepared with chloroform and the active material (C1, C2, and C3) by using a drop and dry method on a conducting indium tin oxide-coated polyethylene terephthalate (PET) film. Dielectric constant measurements were conducted using a parallel-plate configuration on a flexible PET substrate. Each dielectric electrode (C1, C2, and C3) was fabricated on the PET film with a thickness of 80 μm and an active surface area of 15 mm^2^. After deposition films were dried on a hot plate for 30 min, a circular gold electrode was coated on the top side of the film using the physical vapor deposition technique (EMSCOPE SC 500). The materials (C1, C2, and C3) were sandwiched between the conducting PET film (bottom) and the conducting gold-coated (top) electrodes (||-plate configuration). The fabricated film thickness was measured by cross-section microscopy. The material (C1, C2, and C3)-coated PET substrate was fractured to expose the cross section. The sample was mounted edge-on and imaged using an optical microscope (500× magnification). The film thickness was determined as the distance between the film surface and the substrate interface at multiple positions, and an average value (within ±5%) was recorded. The LCR meter recorded the dielectric properties of the thin films (C1, C2, and C3). During the measurement, a small sinusoidal excitation voltage of 100 mV (rms) was applied to ensure the linear dielectric response and to minimize nonlinear effects under zero DC bias conditions. During the dielectric measurement, parallel-plate capacitance (*C*_p_), loss factor (tan δ), and phase (θ) were recorded simultaneously. The real (ε′) and imaginary (ε″) parts of the dielectric constant were extracted by using the relation ε′ = *C*_p_ *d*/ε_0_ *A*, where ε_o_ is 8.85 × 10^–12^ F/m (constant), d is the material thickness, and A is the electrode surface area. The imaginary (ε″) part is calculated from the relation ε″ = ε′ × tan δ.

## Results and Discussion

### Synthesis

The Ni­(II)-derived Schiff base complexes (C1–C3) were synthesized using a one-pot approach without isolation of the ligands (Scheme 1). The synthesis details of the ligands, along with their respective crystal structures, have been previously reported in our work.^35^ To synthesize the complexes, we reacted the corresponding primary amines with 2-hydroxybenzaldehyde in a separate reaction flask using methanol as a solvent in the presence of a catalytic amount of formic acid at room temperature for 1 h (Scheme 1). Subsequently, a methanolic solution of the nickel salt was added to each and stirred for an additional 3 h to obtain the desired Ni­(II) chelate (C1–C3) as a precipitate from the reaction mixture (Scheme 1). Formation of the complexes and the ligands were unequivocally confirmed by single-crystal X-ray diffraction analysis. The structures of the complexes, alongside their color codes, are depicted in Figure 1. The compounds were obtained in moderate yields (50–71%), with colors ranging from green, dark green to brown, respectively (Figure 1). They were found to be air- and moisture-stable, with melting points ranging from 195 to 262 °C. Further details on the characterization of the compounds are provided below.

**Figure 1:**
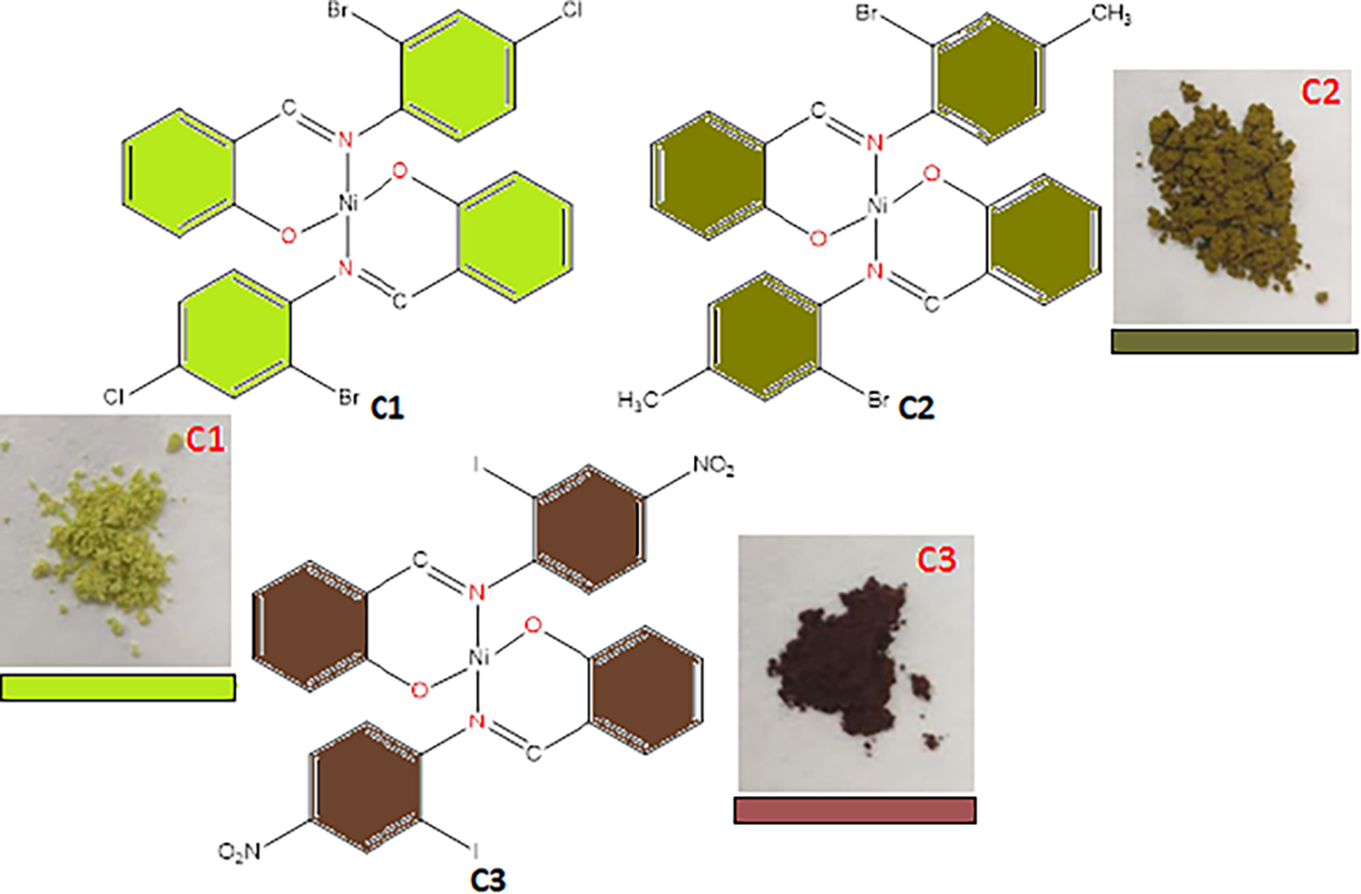
Molecular structures of complexes C1, C2 and C3, alongside the corresponding colors of the isolated solid products.

### Structural Characterization

The synthesized complexes C1–C3 were subjected to various characterization techniques to elucidate their structures. These techniques consist of ^1^H and ^13^C NMR, FTIR, UV–vis, HRMS, and SCXRD analysis. The detailed spectral data obtained from this characterization are presented in the Supporting Information (Figures S2–S16). These spectra provide information that supports the formation of the complexes (C1–C3).

### Nuclear Magnetic Resonance (NMR) Study

In the ^1^H NMR spectra of complexes C1, C2, and C3 (Figures S1, S6, and S11, respectively), the signal corresponding to the hydroxyl group proton, which is typically expected to appear in the downfield region around 11.00–14.00 ppm,^57–59^ was not detected. The absence of this signal suggests deprotonation and subsequent coordination of the nickel ion to the phenolate oxygen, leading to the formation of a nickel–oxygen bond. Additionally, a singlet signal within the range of 8.00–8.50 ppm in the complexes’ spectra indicated the presence of the azomethine (HC=N) proton and affirmed the formation of Schiff base molecules before forming their respective complexes. However, these protons exhibited a downfield shift compared to the free Schiff base ligands documented in the literature.^60,61^ This shift is attributed to the involvement of the azomethine nitrogen in coordination with the nickel ion through a lone pair of electrons. The interaction with the nickel­(II) ion reduces the electron density on nitrogen, causing deshielding of the proton and its appearance in the downfield region. Similar findings have been reported for metal complexes derived from Schiff base ligands.^62,63^ Furthermore, signals corresponding to aromatic protons were observed around 5.6–7.8 ppm in the complexes’ spectra, accounting for all the protons in the compounds. The ^13^C NMR spectra for C1, C2, and C3 (Figures S2, S7, and S12, respectively) display signal peaks corresponding to all the carbon atoms within the compounds. The aromatic carbon atoms were observed in the range of 114–146 ppm, with the carbon atom bonded to the phenolic oxygen appearing further downfield in the spectra due to the influence of the electronegative oxygen atom. Signals from the azomethine (HC=N) carbon atoms were detected in the 164–189 ppm region of the spectra, exhibiting a more downfield shift compared to what would be observed in a free ligand.^64,65^ This shift is a result of the azomethine nitrogen’s involvement in coordination with the nickel ion, forming a Ni–N bond. The data obtained from this study confirmed the successful formation of the complexes, where the ligand acted as a bidentate mononegative ligand coordinated with the nickel ion through the oxygen and nitrogen atoms of the phenolate and hydroxyl moieties.

### Infrared Spectral Study

To investigate the functional groups within the ligands and confirm those involved in coordination with the Ni­(II) ion, the infrared spectra of the complexes (C1–C3) were recorded in the solid state using the ATR method, with the spectra provided in the Supporting Information (Figures S3, S8, and S13). As Schiff base molecules, a crucial diagnostic feature validating a successful reaction is the presence of the azomethine (C=N) bond vibrational stretching frequency.

Typically observed within the range of 1620–1650 cm^–1^ in uncoordinated ligands, this stretching vibrational frequency often shifts to lower values upon coordination with metal ions.^66,67^ In the spectra of the complexes, the vibrational stretching frequency attributed to the azomethine (C=N) bond was within the range of 1595–1600 cm^–1^. This decrease in vibrational frequency results from the coordination of the nitrogen atom to the Ni­(II) ion, leading to a reduction in electron density on the nitrogen and the formation of a nickel–nitrogen bond. Furthermore, the vibrational frequency associated with the hydroxyl group, expected within the 3000–3200 cm^–1^ range, was notably absent.^68^ This absence is a consequence of deprotonation and subsequent coordination of the oxygen atom to the Ni­(II) ion, forming a nickel–oxygen bond. This observation was previously confirmed by NMR spectral studies, which indicated the absence of a hydroxy proton signal and a shift in the azomethine proton signal. Similarly, the presence of peaks related to C–N stretching vibrations observed within the 1432–1438 cm^–1^ range in the spectra of the complexes suggests the presence of the aromatic amine moiety in the ligands.^69,70^ Bands indicating Ni–O and Ni–N stretching vibrations, visible in the region of 400–560 cm^–1^, confirm the coordination of the ligands to the Ni­(II) ion.^71–74^

### UV–Vis Absorption Study

The electronic spectra of the complexes were obtained in a DMSO solution at sample concentrations of 1 × 10^–3^ M using a solution method at room temperature. The spectra of complexes C1–C3, Supporting Information (Figures S4, S9, and S14), reveal systematic differences that correlate with their electrochemical performance in asymmetric supercapacitor coin cells. All complexes show high-energy π→π* (≈232–274 nm) and *n*→π* (≈290–342 nm) bands associated with the azomethine functionality (≈290–342 nm).^75,76^ Crucially, the lowest-energy metal-centered/charge-transfer feature shifts markedly across the series: C3 exhibits a broad, low-energy transition centered at ≈530 nm (≈2.34 eV), C1 at ≈435 nm (≈2.85 eV), and C2 at ≈442 nm (≈2.81 eV). The pronounced red shift and intensity of the low-energy band in C3, whose ligand contains electron-withdrawing nitro groups and heavy iodine atoms, indicate enhanced metal–ligand charge-transfer (LMCT/MLCT) character and a reduced effective optical gap.^77^ This electronic structure favors greater electronic delocalization and improved charge-transfer kinetics at the electrode–electrolyte interface, which in turn increases redox accessibility of the Ni center during cycling. Thus, the optical signatures (stronger low-energy CT absorption and lower-energy d–d/CT bands) provide a straightforward spectroscopic explanation for the observed performance trend C3 > C1 > C2: C3′s stabilized LUMO and enhanced LMCT promote faster electron transport and appreciable specific capacitance, C1 shows intermediate behavior, and C2, bearing electron-donating methyl substituents, exhibits weaker CT character and the least favorable charge-storage properties. The summary of the electronic absorption data is presented in the Supporting Information (Table S1).

### Crystallographic Analysis

The crystal structures of complexes C1 and C2 (Figure 2A,B) show that both compounds crystallize in the monoclinic crystal system, with C1 adopting space group *C*2/*c* and C2 crystallizing in *P2*_1_/*c*. Each structure features a Ni­(II) ion coordinated in a square planar geometry by two phenolic oxygen atoms and two imine nitrogen atoms from the Schiff base ligands, forming the N_2_O_2_ coordination core.

**Figure 2:**
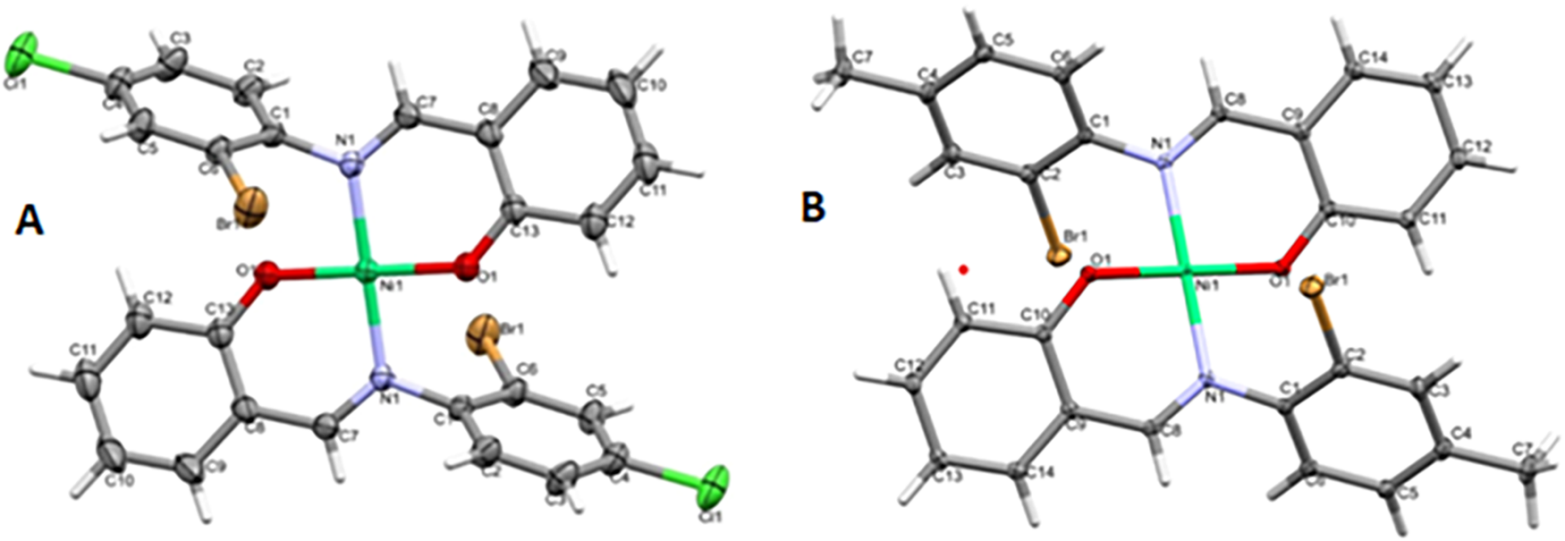
ORTEP diagrams of the molecular structures of complexes (A) **C1** and (B) **C2** with 50% probability ellipsoids for all non-H atoms, while H atoms are represented with the arbitrary radius. Both structures crystallize in the monoclinic crystal system, with C1 in the space group *C*2/*c* and C2 in the space group *P*2_1_/*c*. Each complex exhibits a square planar coordination geometry around the Ni­(II) center. Crystallographic details are available in the Supporting Information in Table S2.

In C1, the Ni­(II) center lies on a crystallographic inversion center, resulting in a symmetric arrangement, with each ligand adopting a bidentate mode. The Ni–O and Ni–N bond lengths fall within expected ranges for square planar Ni­(II) complexes, with minimal distortion from the ideal geometry. The symmetric unit of C2, which is a methyl analogue of C1, contains one molecule of the complex. Similar coordination behavior is observed, with Ni–O and Ni–Ni bond distances comparable to those in C1, suggesting negligible electronic effects from the methyl substituents. The planar geometry around the Ni­(II) centers in both complexes is further supported by the trans arrangement of the donor atoms and the near-ideal cis angle of approximately 90°. These structural similarities highlight the robustness of the coordination environment, while the differences in the space group and packing may influence crystal stability and intermolecular interaction.

An analysis of the crystal packing of C1 reveals stabilization through nonclassical hydrogen bonding interactions, specifically C5–H5···Br1 (3.864 Å) and C2–H2···C8 (2.877 Å), forming a diagonal array (Figure 3A). These intermolecular interactions contribute to the overall structural stability of the complex. The complex crystallizes in the monoclinic *C*2/*c* space group, with the following unit cell parameters: *a* = 21.3488(6) Å, *b* = 6.7330(2) Å, *c* = 18.0467(5) Å, and β = 97.030(1)°, yielding a unit cell volume of 2574.56(13) Å^3^. Crystallographic refinement resulted in *R*_1_ = 0.0270 and w*R*_2_ = 0.0726 for observed data (*I* ≥ 2σ­(I)), demonstrating high-quality structural determination. The structure exhibits a goodness-of-fit (GoF) value of 1.089, with minimal residual electron density peaks (0.63 and −0.61 e Å^–3^), further confirming the accuracy of the refinement (Table S2).

**Figure 3:**
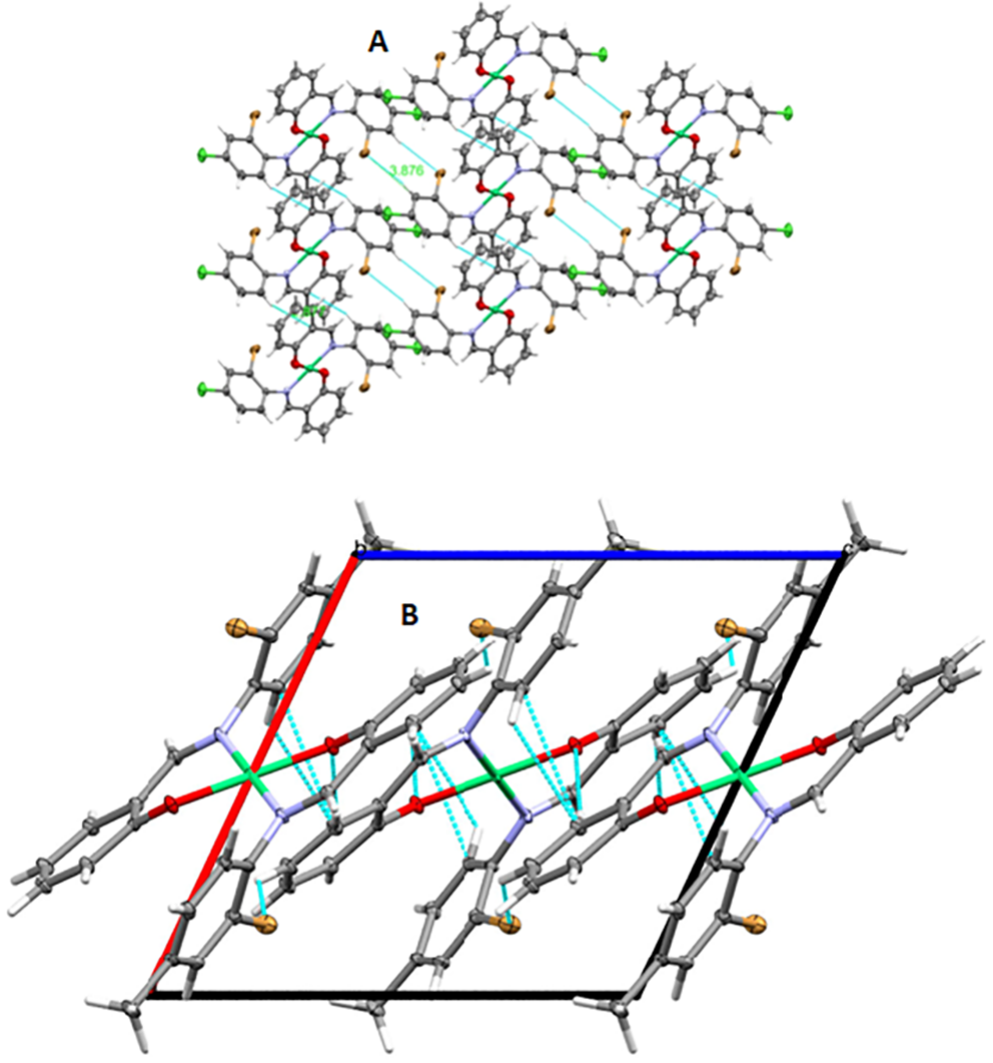
Crystal packing of the molecule observed along the *b*-axis in the solid-state crystal structures of **C1** (A) and (B) **C2**; Atom representation: H atoms are illustrated as capped cylinders. Atom color code: white (H), gray (C), light blue (N), light green (Ni), red (O), dark green (Cl), and brown (Br).

In the solid state of C2, the molecular packing is stabilized predominantly by π–π stacking interactions between aromatic rings, forming extended layers along the crystallographic *b*-axis (Figure 3B). The centroid-to-centroid distances and slippage suggest efficient overlap, contributing to the dense packing. Additional weak Br···π or C–H···O interactions may further stabilize the supramolecular architecture. The crystallographic architecture emphasizes the role of directional noncovalent interactions in governing solid-state stability and highlights the potential of such complexes in materials and coordination chemistry applications. Together, the characterization techniques confirm the successful isolation of the complexes and their corresponding chemical structures and purity.

### Electrochemical Evaluation of C1, C2, and C3 Electrodes under 2 M KOH Electrolytes

The electrochemical performances of the three different Ni-based Schiff base ligands C1, C2, and C3 were investigated in a three-electrode system in 2 M KOH. Cyclic voltammetry (*C–V*) and galvanostatic charge–discharge (GCD) analyses of the fabricated C1, C2, and C3 electrodes are presented in Figure 4A,B, respectively. The *C–V* curves were recorded at a scan rate of 20 mV·s^–1^ within a potential range of 0–0.6 V. Each electrode’s *C–V* curve (C1, C2, and C3) exhibits a primary redox peak, corresponding to Ni^2+^/Ni^3+^ transition, and a secondary low-intensity diffuse peak linked to stepwise electron transfer or the phase change reaction of nickel species within the structure under alkaline conditions. The *C–V* and GCD curves of the bare nickel foam (NF) under identical conditions are plotted in the inset of Figure 4A,B, respectively. No background subtraction was applied during the electrochemical method; instead, the current response of the bare NF was independently measured and verified to be negligible under the operating conditions used for the active materials. Previous studies have reported that the Ni-coordinated Schiff ligand facilitates the electron transfer process and redox activity via π-bond interaction.^31,42,78,79^ In the present systems, *C–V* curves exhibit similar behavior, which is attributed to the mass transfer reactions at the electrode (nickel center)–electrolyte interfaces.

**Figure 4:**
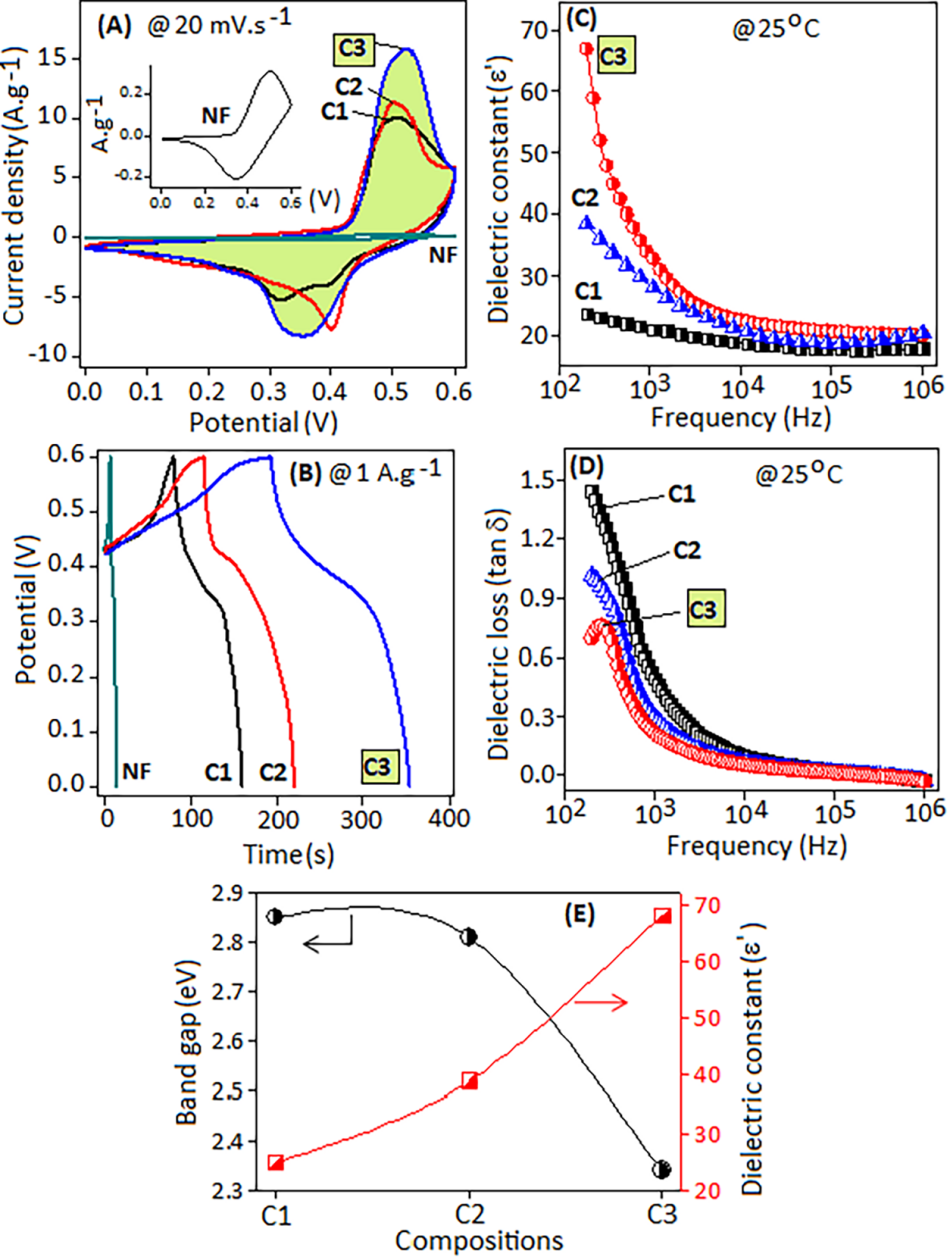
Comparison of (A) cyclic voltammetry curves at a scan rate of 20 mV·s^–1^ and (B) charge–discharge curves for bare Ni-foam (NF), C1, C2, and C3 electrodes. Inset (A) shows the expanded view of NF contribution. (C) Variation of dielectric constant and (D) dielectric loss of C1, C2, and C3 thin-film devices under the applied frequency (200 Hz to 1 MHz). (E) Variation of dielectric constant (ε′) versus optical band gap of C1, C2, and C3 systems.

Among the three electrodes, the C3 electrode displays a higher current response, improved redox behavior (green color) at a scan rate of 20 mV·s^–1^, and long-term charge–discharge capability at a current density of 1 A·g^–1^. The improved performance of the C3 electrode can be ascribed to the coordinated iodide ion, which participates in the charge-transfer process and influences the electron density around the Ni center. This stabilizes the π-bond interactions and enhances interatomic polarizability. Additionally, the iodide ion exhibits lower electronegativity compared to the halide ions present in the C1 (chloride) and C2 (bromide) systems. These factors collectively contribute to the better electrochemical behavior of the C3 material, confirming its potential for use in supercapacitors and energy storage devices. The dielectric constant (ε′) of C1, C2, and C3 thin-film devices was investigated at room temperature over a frequency range of 200 Hz–1 MHz, as shown in Figure 4C. The C3 device exhibits a dielectric constant (ε′) value of ∼68 at 200 Hz, with the value decreasing as frequency increases. The variation in ε′ is more pronounced at lower frequencies (below 10 kHz), which is attributed to space charge and ionic polarization within the material. The space charge polarization accumulated at material interfaces or boundary regions, and ionic polarization linked to mobile defects and residual ions in the Ni–Schiff complexes. At higher frequencies, the induced polarizations cannot keep pace with the frequency changes, resulting in a decrease in the dielectric constant. In the C3 system, the higher polarizability of the iodide (I^–^) ion enhances ionic polarization, thereby improving the dielectric constant performance compared to the C1 and C2 systems. The high polarizability of the iodide ion also amplifies the dielectric loss peak (tan δ), as observed in the C3 device (Figure 4D). Conversely, the C1 and C2 devices show higher dielectric loss due to the increased mobility and stronger electrostatic interactions of the smaller halide ions (Cl^–^), which accumulate at the interface region. These properties lead to significant energy dissipation within the C1 and C2 devices, resulting in lower dielectric constant values. The difference in ε′ values among C1–C3 is therefore interpreted as a qualitative indicator of relative electronic polarizability. While a higher ε′ is consistent with enhanced charge separation and improved ionic accommodation, these dielectric values do not quantitatively account for the measured pseudocapacitive response. Instead, they provide a supportive context for understanding the electronic environment of the complexes.

The dielectric constant (ε′) and optical band gap (eV) provide complementary information about how the electronic structure of the complexes influences charge storage, as illustrated in Figure 4E. A higher dielectric constant enhances the material’s ability to polarize under the applied electric field, facilitating ion accumulation and improving charge separation. Conversely, a smaller band gap reflects increased ligand-to-metal charge-transfer activity and higher electronic polarizability, which can promote faster redox kinetics and improved conductivity. Together, these parameters offer a qualitative indication of how the molecule’s electronic environment governs pseudocapacitive behavior. While they do not establish a direct quantitative predication of capacitance, they assist rationalize the observed electrochemical trend across the C1, C2 and C3 systems.^80^

The *C–V* and GCD profiles of the C3 electrode were further examined at various scan rates (5 to 50 mV·s^–1^) and current densities (1 to 8 A·g^–1^) within a potential window of 0–0.6 V, as illustrated in Figure 5A,B, respectively. With increasing scan rates, the area under the *C–V* curve expands and the peak intensity shifts toward higher potentials, indicating a reversible electrochemical reaction in the electrode. The GCD curves exhibit a nonlinear pattern with a plateau-like feature during the discharge phase, corresponding to pseudocapacitive properties. In the C3 electrode, the redox process involves the reversible interconversion of Ni^2+^ ⇌ Ni^3+^ ions within the Schiff ligand network.^42,79^

**Figure 5:**
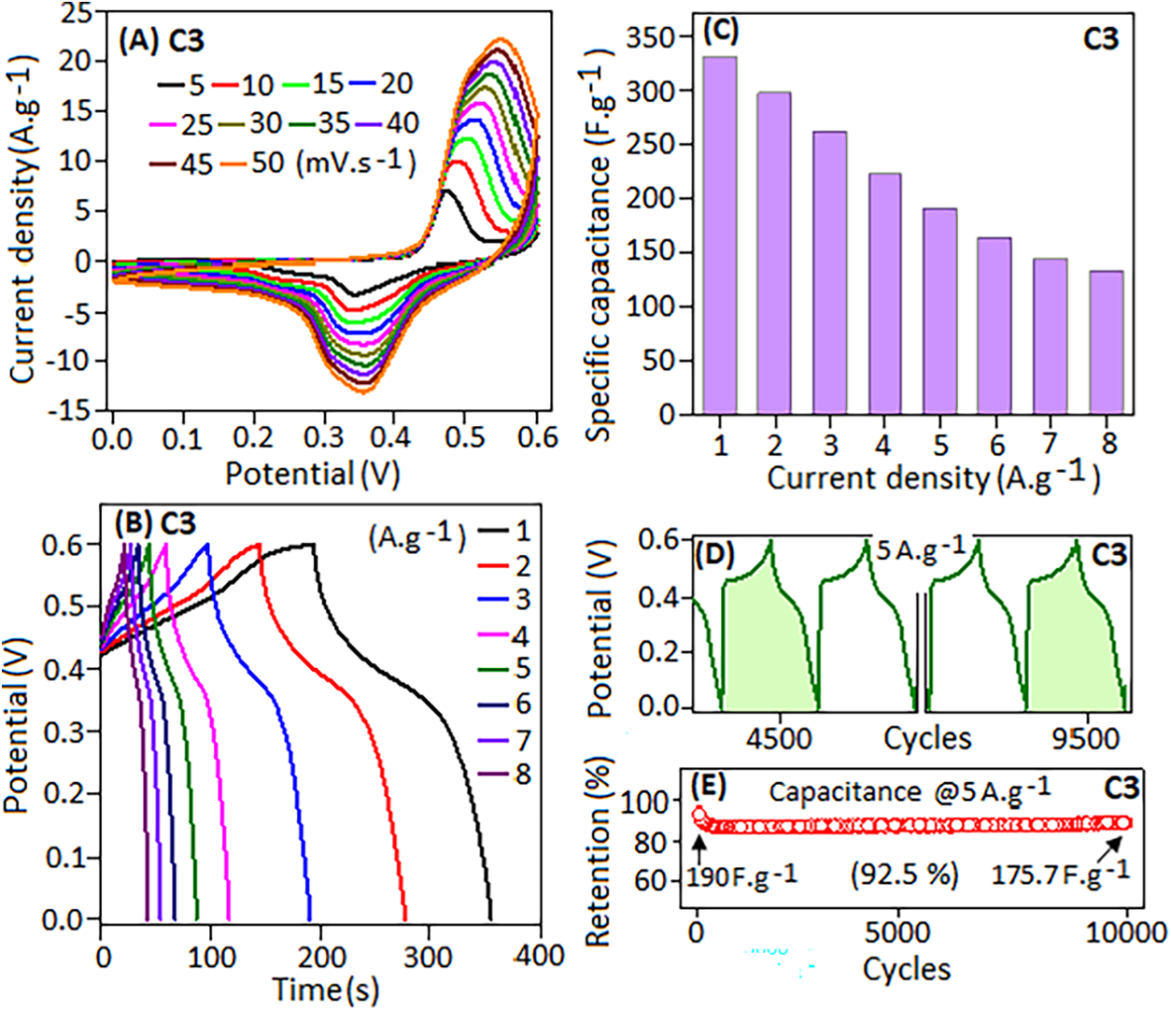
(A) *C–V* curves of the C3 electrode at different scan rates from 5 to 50 mV·s^–1^. (B) GCD profile of the C3 electrode at different current densities from 1 to 8 A·g^–1^. (C) Histogram plot of specific capacitance values with respect to different current densities. (D) Cycling stability of the GCD profile after 4500 and 9500 (green color region) cycles (highlighted region) and (E) capacitance retention (%) of the electrode at 5 A·g^–1^ over 10,000 cycles.

This process is facilitated by the diffusion of OH^–^ ions from the electrolyte to the electrode surface during oxidation and their return to the electrolyte during reduction. The reaction mechanism for the C3 (Ni-based Schiff base ligand) electrode can be represented as follows:

I $${\mathrm{Ni}}^{2+}-\mathrm{Schiff}\;\mathrm{base}+{\mathrm{OH}}^{-}⇌{\mathrm{Ni}}^{3+}-\mathrm{Schiff}+e^{-}$$

The Schiff base ligand stabilizes the Ni^2+^ state through its π-conjugation and electron-donating effects, which facilitate charge and delocalization and lower the energy barrier for oxidation. When the potential is reversed, the Ni^3+^ state transitions back to the Ni^2+^ state, a process that is typically reversible under standard electrochemical conditions in KOH.

Additionally, in an alkaline medium, the nickel center can form surface-bound species such as Ni­(OH)_2_ (Ni^2+^ state) and NiOOH (Ni^3+^ state), as described by the following reaction:

II $${\mathrm{Ni}}^{2+}-\mathrm{Schiff}+{2\mathrm{OH}}^{-}\left({\mathrm{Ni}\left(\mathrm{OH}\right)}_{2}\right)⇌\mathrm{NiOOH}+H_{2}O{+e}^{-}$$

A secondary low-intensity redox peak is clearly visible in all three (C1, C2, and C3) electrodes, which are associated with the structural changes in the (β/γ)-polymorphs of NiOOH and stepwise electron transfer between polymorphs. The corresponding cathodic peak appears on the reverse scan, often at different potentials due to the kinetics. The appearance of a secondary redox peak in the reverse scan rate is attributed to the slower formation and accumulation of NiOOH polymorphs during anodic sweep, which is then rapidly and collectively reduced upon the cathodic sweep, producing a sharper reduction peak. At high scan rates, these redox peaks broaden and merge due to the combined effect of charge-transfer kinetics and overlapping multistep Ni redox transitions. To validate the above reaction mechanism ([Disp-formula eq1] and 2), the X-ray photoelectron spectroscopy (XPS) analysis were performed separately on the pristine C3 sample and the post-cycle sample (after cyclic voltammetry), as shown in Figure S16a (Supporting Information). The elements Ni, C, N, O, and I were clearly detected in the survey spectra of the C3 material. The high-resolution Ni 2p emission spectrum of the pristine C3 system shows two strong peaks positioned at ∼855.3 eV (Ni 2p_3/2_) and ∼873.1 eV (Ni 2p_1/2_), corresponding to the Ni^2+^ oxidation state. The spectrum also contained two additional peaks, shake-up (satellite) peaks of nickel at ∼862.8 eV and ∼880.2 eV, as highlighted in Figure S16b (Supporting Information). During the cyclic voltammetry of the pristine C3 system, the Ni^2+^ state (Ni­(OH)_2_) undergoes a reversible Ni^3+^ transition state (NiOOH) under the KOH medium, which validates the presence of two additional diffuse peaks in the Ni 2p spectrum positioned at ∼860.1 and ∼874.3 eV, corresponding to the Ni^3+^ oxidation state, and the satellite peak intensity diminishes the as Ni^3+^ component grows in the system. After cycling, the slight shift toward higher binding energy and variation in the intensity ratio of Ni^2+^/Ni^3+^ confirm the reversible redox transition Ni­(OH)_2_ ↔ NiOOH during the charge–discharge process. This transformation is responsible for the pseudocapacitive behavior observed in the GCD profiles. The broadening of peaks and the reduction in satellite intensity after long-term cycling suggest partial surface oxidation/hydration and restructuring of the near-surface layer. These changes improve the ionic accessibility and contribute to enhanced electrochemical activity in the initial cycles. The modification in the peak shape and position in the XPS spectra thus reflects the formation of an electrochemically active surface layer and a reversible redox process rather than degradation. This correlates with the stable capacitance retention observed during 10,000 charge–discharge cycles, confirming the structural adaptability of the C3 electrode to continuous redox reactions in alkaline media.

The specific capacitance (*C*_p_) values of the electrode were calculated from the GCD profile at different current densities ranging from 1 to 8.0 A·g^–1^ by using the following equation

1 $$C_{p}=I\times \Delta t/m\times \mathrm{dV}$$

where *I* is the current density, *dV* is the potential window, and Δ*t* is the discharge time (s) at a particular current density (A·g^–1^).

Figure 5C presents a histogram of specific capacitance (*C*_p_) values at varying current densities. At a current density of 1 A·g^–1^, the electrode achieves a maximum *C*_p_ value of ∼330 F·g^–1^, which decreases to about 133.5 F·g^–1^ at 8 A·g^–1^. This reduction at higher current densities is attributed to the limited interaction of ions with the electrode surface due to rapid charge-transfer kinetics. Conversely, at lower current densities, both the inner and outer active sites of the electrode are accessible for the charge-storage process, resulting in higher capacitance values. The charge–discharge stability of the fabricated electrode was evaluated over 10,000 cycles at a current density of 5 A·g^–1^, without any significant distortion in the charge–discharge profile, which is highlighted (color region) in Figure 5D. After 10,000 cycles, the electrode retained approximately 92.5% of its initial specific capacitance (∼190 F·g^–1^), corresponding to ∼175.5 F·g^–1^, as illustrated in Figure 5E.

### Dielectric Behavior Study of the C3 Electrode

The electrochemical kinetics of the C3 electrode was analyzed from the *C–V* curves. In cyclic voltammetry, the variation of peak current (*I*_p_), both anodic and cathodic, is related to the scanning rate (ν) by the relation *I*_p_ = *a*ν*^b^*, where “*a*” is an adjustable parameter and the slope of the log (*I*_p_) vs log (ν) estimates the value of “*b*”. For a diffusion-controlled process, the value of *b* = 0.5, while for a capacitive-controlled process, *b* = 1. The diffusion behavior inside the electrode is further confirmed when a linear relationship is plotted between peak current (*I*_p_) for both anodic and cathodic versus the square root of scan rate (ν^1/2^) (Figure 6A). The inset of Figure 6A shows a linear fit between log­(*I*) vs log­(*v*) curves at a fixed potential of 0.55 V for the C3 electrode, yielding a *b* value of ∼0.58, which suggests that the diffusion-controlled process dominates in charge-storage kinetics. However, during cyclic voltammetry, diffusion-controlled currents (inner-surface contributions) dominate at lower scan rates, while capacitive currents (outer-surface contributions) become more significant at higher scan rates. These phenomena correspond to Faradaic and non-Faradaic contributions, respectively.^81,82^ At lower scan rates, the slower potential change allows sufficient time for electroactive species to diffuse into the electrode material. Conversely, at higher scan rates, rapid potential changes limit the process to interactions at the outer surface of the electrode.

**Figure 6:**
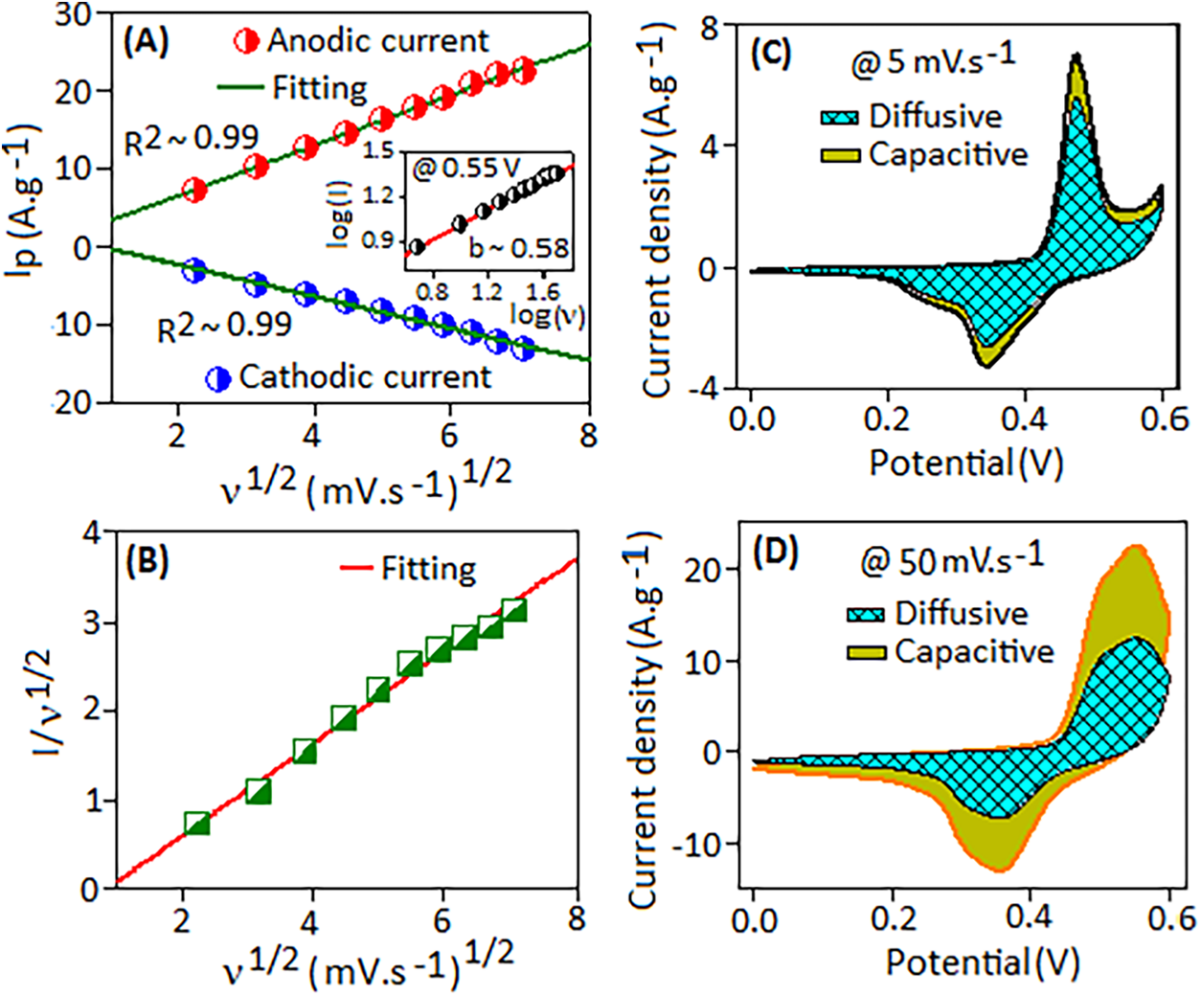
(A) Linear relation of the anodic and cathodic peak current *I*_p_ versus *n* ^1/2^, and the inset shows a linear fit of the current (*I*) versus scan rate (ν) in a log scale. (B) Linear fitting of *I*/*n* ^1/2^ versus *n* ^1/2^ at 0.55 V according to eq 4. Diffusive and capacitive contributions extracted from the experimental *C–V* curves at 5 mV·s^–1^ (C) and 50 mV·s^–1^ (D) scan rates.

During electrochemical operation, the electrode’s reaction kinetics involves a combination of diffusion-controlled and capacitive-controlled charge-storage mechanisms, which together influence the supercapacitor’s performance.^82,83^ According to Dunn’s method, the current contribution in the *C–V* profile at a fixed potential can be expressed as the sum of diffusion- and capacitive-controlled processes using the following equation:^84^

2 $$I=k_{1}v+k_{2}v^{1/2}$$

In the above relation, *k*_1_ *v* and *k*_1_v^1/2^ correspond to the capacitive- and diffusion-controlled contributions in the electrochemical process, respectively. The above Dunn relation and a linear fitting of *I*/ν^1/2^ = *k*_1_ν^1/2^ + *k*_2_ at a fixed potential (0.55 V) are rearranged when *I*/v^1/2^ versus v^1/2^ is plotted, as illustrated in Figure 6B. The fitting provides the values of *k*_1_ (slope) and *k*_2_ (intercept). From the values of *k*_1_ and *k*_2_, the absolute and percentage values of surface-capacitive and inner-diffusive contributions to the C3 electrode were estimated at different scan rates. The capacitive contribution (olive color region) and diffusive contribution (shaded region) at low (5 mV·s^–1^) and high (50 mV·s^–1^) scan rates are plotted in Figure 6C,D, respectively.

### Electrode Electrochemistry Study

The variation in the diffusive and capacitive contributions (%) of the different scan rates is illustrated in Figure 7A. At a low scan rate of 5 mV·s^–1^, the electrode exhibited ∼80% diffusive contribution, which reduced to about 58% at a higher scan rate of 50 mV·s^–1^. This observation suggests that the ion diffusion predominantly governs the electrochemical redox process at lower scan rates. As the scan rate increases, the capacitive contribution (outer surface interaction) gradually becomes more significant, while the diffusion-controlled contribution diminishes. At higher scan rates, the insufficient time for the electrolyte ions to penetrate the inner sites of the active material leads to a decreased diffusion process and a dominance of surface or near-surface interactions in charge storage. These results collectively support a hybrid charge-storage mechanism in C3, where fast surface redox reactions contribute substantially to pseudocapacitive, while a measurable diffusion-controlled component reflects ion transport within the electrode structure. A comparative study, shown in Figure 7B, highlights the acceptable performance of the C3 electrode consistent with other reported Schiff-based systems as an electrochemical supercapacitor.^43,45,78,79,85–88^ A detailed comparison of specific capacitance values, electrolyte conditions, and retention performance between the present C3 electrode and previously reported Schiff base materials is summarized in Table 1.

**Figure 7:**
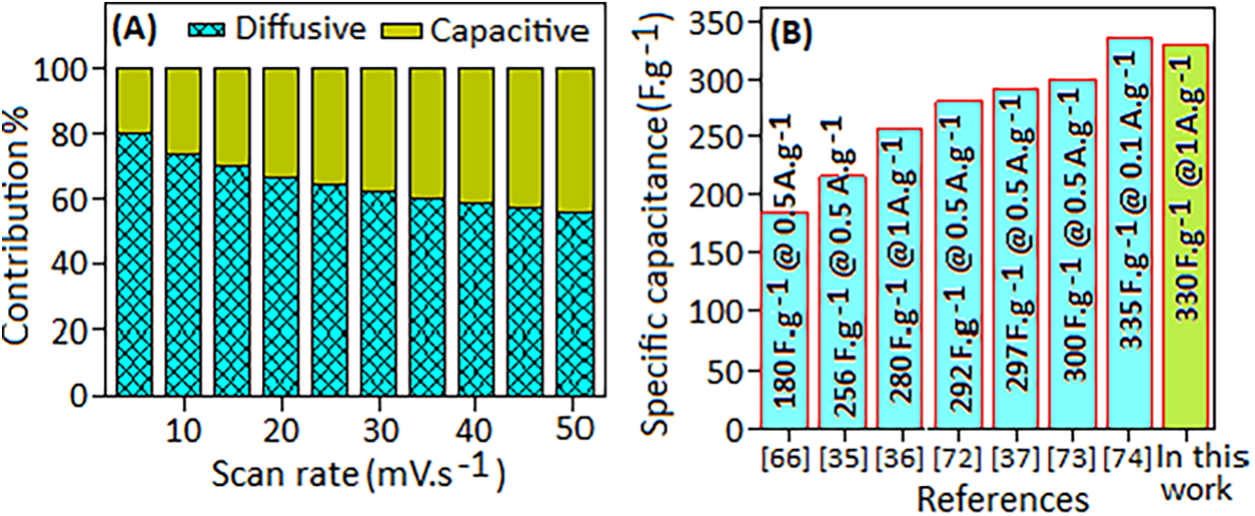
(A) Bar graph plots of diffusive and capacitive contributions (%) versus different scan rates of the electrode. (B) Comparison of specific capacitance values of the present C3 electrode with other reported carbon/Schiff base-based electrode materials.^43,45,78,79,85–88^

**Table 1:** Comparison of Specific Capacitance Values of the Fabricated C3 Electrode with Various Carbon-Based and Schiff Base-Based Electrode Systems^a^

**carbon/Schiff base-based electrode**	**electrolyte condition**	**specific capacitance**(F·g^**–1**^ **)**	**capacitance retention**(%)	**refs**
3D porous carbon on a Schiff base polymer	LiTFSI	180 F·g^–1^ @ 0.5 A·g^–1^	93.8% @ 10,000 cycles	^35^
viologen–Schiff base polymer	3 M KOH	256 F·g^–1^ @ 0.5 A·g^–1^	95% @ 3000 cycles	^37^
cross-linked scaffold	6 M KOH	280 F·g^–1^ @ 1 A·g^–1^	58% @ 10,000 cycles	^66^
nitrogen/oxygen-codoped Schiff base polymer	6 M KOH	292 F·g^–1^ @ 0.5 A·g^–1^	96% @ 2000 cycles	^71^
N-doped carbon derived from a poly-Schiff base	1 M Na_2_SO_4_	297 F·g^–1^ @ 0.5 A·g^–1^	89.4% @ 10,000 cycles	^72^
N-doped carbon aerogels extracted from a Schiff polymer	1 M H_2_SO_4_	300 F·g^–1^ @ 0.5 A·g^–1^	98% @ 5000 cycles	^73^
graphene-coupled Schiff base polymer	6 M KOH	335 F·g^–1^ @ 0.1 A·g^–1^	90% @ 10,000 cycles	^74^
nickel(II) complex of Schiff base-derived from a 2-iodo-4-nitroaniline-based electrode (C3)	2 M KOH	330 F·g^–1^ @ 1 A·g^–1^	92.5% @ 10,000 cycles	in this work

a LiTFSI: lithium bis­(trifluoromethanesulfonyl)­imide.

### Asymmetric AC//C3 Supercapacitor Device for Energy Harvesting Performance

An asymmetric supercapacitor coin cell is a device in which the positive electrode (cathode) and negative electrode (anode) are made of different materials with different electrochemical properties. Different electrodes have different stability and redox potentials, which can extend the operating voltage window in an aqueous medium. Asymmetric combination merges their strengths, pushes the voltage window, and increases the energy density for energy storage applications. In this view, the energy storage performance of the C3 electrode was evaluated in the context of a supercapacitor device. The device was assembled as an asymmetric coin cell, designated AC//C3, using activated carbon, AC (negative electrode, anode), and C3 (positive electrode, cathode) under the 2 M KOH electrolyte. A schematic figure of the coin cell structure and its components is shown in Figure 8A. Prior to device assembly, the electrochemical performance of the AC electrode was tested as a three-electrode system within a potential range from −1.0 to 0 V in 2 M KOH. The *C–V* performances of the AC and the C3 electrodes are presented in Figure 8B, recorded at a scan rate of 20 mV·s^–1^ over a potential window from −1.0 to 0.6 V. Additionally, the GCD profiles of AC and C3 electrodes at a current density of 1.0 A·g^–1^ are compared in the inset of Figure 8B. The *C–V* curves for the AC electrode exhibit a quasi-rectangular shape, indicating typical capacitive behavior, with a specific capacitance of approximately 80.6 F·g^–1^ (Δ*V*_–_ = 1 V) at a current density of 1.0 A·g^–1^. In comparison, the C3 electrode offers a significantly higher specific capacitance of ∼330 F·g^–1^ (Δ*V*_*+*_ = 0.6 V) at the same current density. Using these values, the mass ratio of ∼0.40 is obtained, with an estimated total electrode mass of ∼5.6 mg (AC and C3), for the asymmetric device. The *C–V* curves of the AC electrode (−1.0 to 0 V) and the C3 electrode (0 to 0.6 V) recorded at 20 mV·s^–1^ suggest a stable potential window of 1.6 V for the device. Extending the voltage to 1.7 V results in a distortion of the *C–V* curve shape and a sudden rise in current, as shown in Figure S17 (Supporting Information).

**Figure 8:**
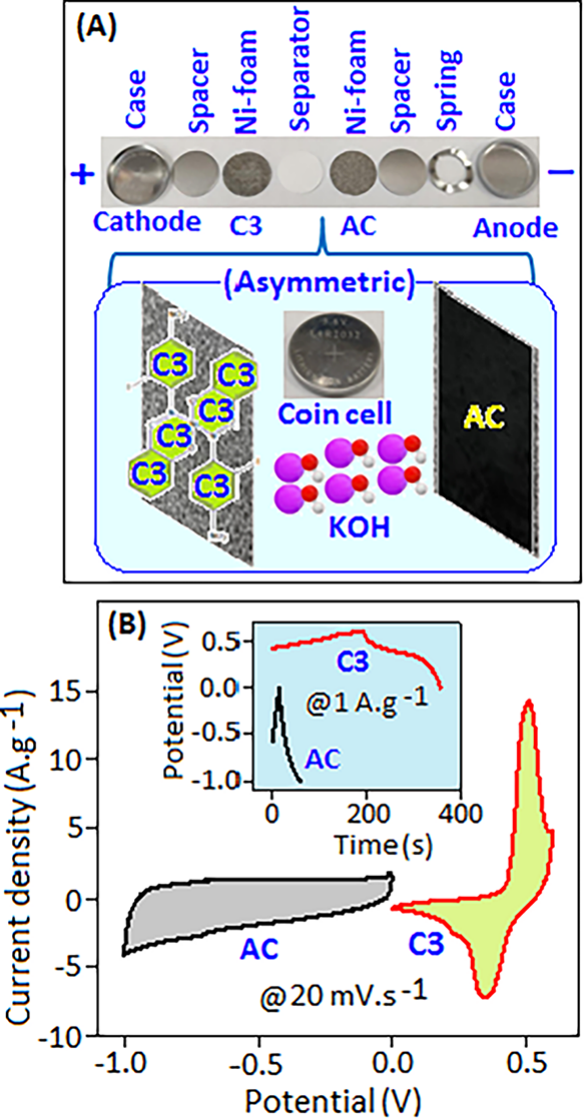
(A) Schematic diagram of an asymmetric AC//C3 supercapacitor coin cell. (B) *C–V* curves of activated carbon (AC, negative electrode, Δ*V* = −1.0 to 0 V) and C3 (positive electrode, Δ*V* = 0–0.6 V) at a scan rate of 20 mV·s^–1^, and the inset highlights the CD pattern of the AC and C3 electrodes at 1 A·g^–1^.

Therefore, the *C–V* performances of the device were evaluated at a potential of 1.6 V across various scan rates ranging from 10 to 100 mV·s^–1^, as shown in Figure 9A. The *C–V* curves retained their shape without significant distortion, even at higher scan rates, indicating stable electrochemical behavior. The GCD profiles of the device, measured at current densities ranging from 0.5 to 4.0 A·g^–1^, are shown in Figure 9B. The specific capacity (*Q*_s_) of the device was determined from the GCD curves using eq 5.

3 $$Q_{s}=I\times \Delta t/3.6\times m$$

where *I* is the current, m is the total mass of the active electrode (*g*), and Δ*t* is the discharge time (s) at a particular current density (A·g^–1^).

**Figure 9:**
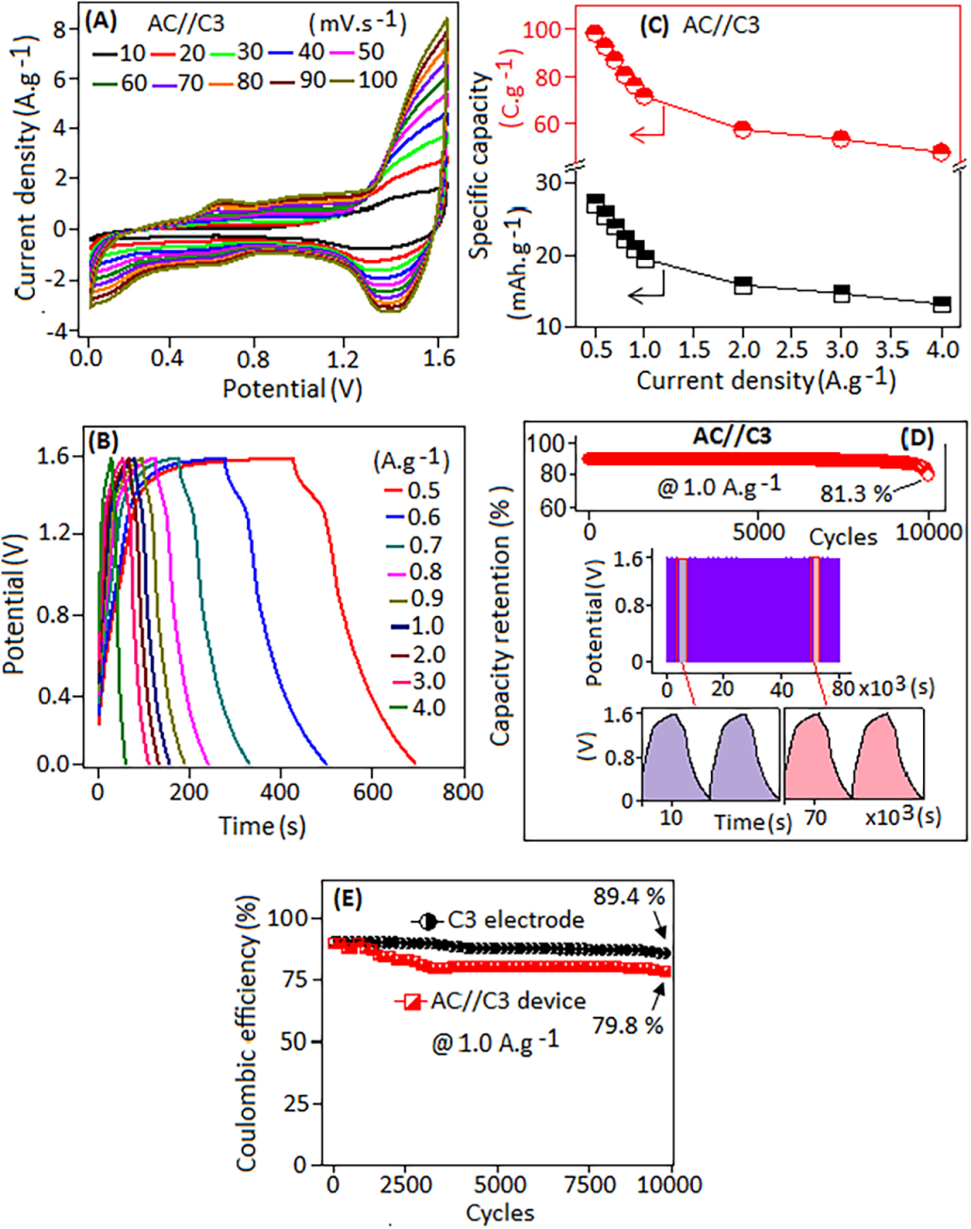
(A) *C–V* curves of the AC//C3 device at different scan rates ranging from 10 to 100 mV·s^–1^ under potentials of 0–1.6 V. (B) GCD pattern of the device at different current densities from 0.5 to 4.0 A·g^–1^. (C) Variation of specific capacity values at different current densities. (D) Capacity retention (%) of the device recorded at 1.0 A·g^–1^ after 10,000 cycles, and the expanded view of the GCD profile at the initial cycle and at the end cycle (color regions). (E) Coulombic efficiency (%) of the C3 electrode and device recorded at 1.0 A·g^–1^ after 10,000 cycles.

The extracted specific capacity (*Q*_s_) values at different current densities (0.5 to 4 A·g^–1^) are plotted in Figure 9C. The specific capacity (C·g^–1^) values were extracted by using the total active mass (negative and positive, 5.6 mg) of the AC//C3 device. At a current density of 0.5 A·g^–1^, the device achieved a *Q*_s_ value of ∼98.3 C·g^–1^ (∼61.4 F·g^–1^ or ∼27.3 mAh·g^–1^), which decreased to ∼47.6 C·g^–1^ (∼29.7 F·g^–1^ or ∼13.2 mAh·g^–1^) at a higher density of 4.0 A·g^–1^. The long-term cycling stability of the device was evaluated over 10,000 cycles at a current density of 1.0 A·g^–1^, as shown in Figure 9D. After 10,000 cycles, the device retained ∼80.3% (∼57.4 C·g^–1^ or ∼35.9 F·g^–1^) of its initial charge (∼71.6 C·g^–1^or ∼44.7 F·g^–1^), with no significant distortion in the charge–discharge profile (color region), as highlighted in Figure 9D. These results demonstrate the reasonable long-term electrochemical stability and durability of the fabricated device. The Coulombic efficiency η_c_ (%) values of the fabricated C3 electrode and asymmetric AC//C3 device were extracted by using the relation η_c_(%) = *t*_d_/*t*_c_, where *t*_c_ and *t*_d_ are the charging and discharging times, respectively. The values of η_c_ (%) of the C3 electrode and the device were extracted at a current density of 1.0 A·g^–1^ (Figure 9E). The result shows that the C3 electrode retained ∼89.4% Coulombic efficiency in a three-electrode configuration due to the controlled potential window and the minimized parasitic reaction on the single working electrode. However, when assembled into an asymmetric (AC//C3) device, the Coulombic efficiency decreased to ∼79.8% after 10,000 cycles, which is due to the additional electrochemical, kinetic, and interfacial complexities. Figure S18 (Supporting Information) highlights the photographs of the dismantled coin cell components such as casing, gasket, separator, spring, both spacers, and both electrodes after 10,000 cycles. The stainless steel casing remained intact without visible corrosion. The gasket exhibited no abnormal swelling or chemical degradation, and the separator retained its structural integrity. The cell was operated at room temperature, maintaining a potential of 1.6 V, with no traces of gas evolution inside the cell during disassembly.

The energy (*E*) density and power (*P*) density of the asymmetric AC//C3 device were estimated from the nonlinear charge–discharge profile (GCD), by an integral approach method using eqs 6 and 7, respectively. During the measurement, a constant discharge current, *I* (A), is applied in the device, and the voltage trace *V­(t)* (*V*) is recorded during the discharge time (*dt*), from *t*_1_ (start) to *t*_2_ (cutoff), according to the following relation

4 $$E=\int_{t1}^{t2}V\left(t\right)\times I\times \mathrm{dt}\left(\mathrm{Joule}\right),\;\mathrm{and}\;E=\frac{1}{3.6\times m}(1\times \int_{t1}^{t2}V\left(t\right)\times \mathrm{dt})\;(W\cdot h\cdot kg^{-1})$$

5 $$P=\frac{1}{\Delta t}\times \left(E\times 3600\right)$$

where ∫_*t*_1__^*t*_2_^ *V*(*t*) × *dt* is the area under the curve, *m* is the mass of the device (in kg), and *V* is the applied potential. Energy and power density values were calculated from the uncompensated galvanostatic charge–discharge traces. The uncompensated values include the instantaneous voltage loss at the start of the discharge (*iR*-drop) and therefore reflect device performance without electronic correction.

The asymmetric AC//C3 device demonstrated an energy density of ∼21.8 Wh·kg^–1^ with a power density of 378.3 W·kg^–1^ at a current density of 0.5 A·g^–1^. At a higher current density of 4 A·g^–1^, the device achieved a power density of ∼1089 W·kg^–1^ while maintaining an energy density of 10.6 Wh·kg^–1^, as shown in Figure 10A. The extracted energy and power density values of the present asymmetric cell (AC//C3) shows comparable performance than some of the previously reported Schiff base-based supercapacitor devices such as hierarchical porous carbon nanoparticles (7.64 Wh·kg^–1^, 250 W·kg^–1^ at 0.5 A·g^–1^),^89^ nitrogen-rich porous carbon nanosheets (9.2 Wh·kg^–1^, 500 W·kg^–1^ at 1.0 A·g^–1^),^90^ polymer-supported nitrogen/oxygen-codoped porous carbons (11.3 Wh·kg^–1^, 103 W·kg^–1^ at 0.2 A·g^–1^),^86^ nitrogen/oxygen-codoped hierarchical porous carbon networks (31.7 Wh·kg^–1^, 562.5 W·kg^–1^ at 0.5 A·g^–1^),^78^ nickel-doped MOF (21 Wh·kg^–1^, 302 W·kg^–1^ at 0.5 A·g^–1^),^91^ and Schiff-based Ni–terephthalaldehyde (Ni–OTTP) complex (34.5 Wh·kg^–1^, 613.2 W·kg^–1^ at 0.5 A·g^–1^).^31^

**Figure 10:**
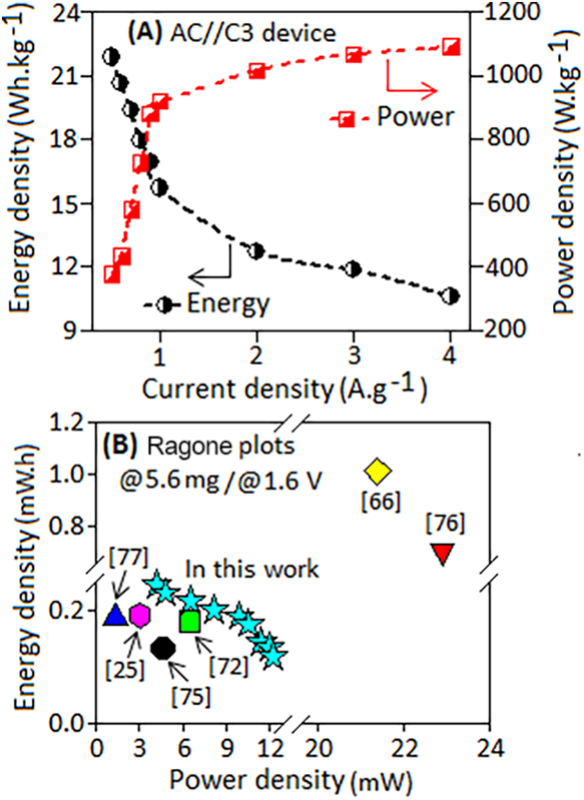
(A) Variation of the energy density and power density of the AC//C3 device at different current densities ranging from 0.5 to 4.0 A·g^–1^. (B) Ragone plot of the present AC//C3 device in comparison with other reported Schiff base ligand-based supercapacitor devices.^31,78,86,89–91^

The total active mass (5.6 mg) and the same potential window (1.6 V) were used to normalize the energy and power density values of the present asymmetric AC//C3 device and previously reported Schiff-based systems (excluding the *iR*-drop in the calculation) at a current density of 0.5 A·g^–1^, enabling a direct and quantitative comparison as illustrated in the Rangon plot (Figure 10B). For a better explanation, two worked examples of energy and power density calculations for asymmetric devices are also included in the Supporting Information. The corresponding values of energy density, power density, and applied potential are also listed in Table 2.

**Table 2:** Recalculation of the Energy Density and Power Density of the AC//C3 Asymmetric Device with Other Reported Schiff-Based Devices Using the Same Active Mass (5.6 mg), the Same Discharge Window (1.6 V), and the Same Current Density (0.5 A·g^–1^)­^a^

**Schiff-based supercapacitor devices optimized @**5.6 mg, @ 1.6 V, and @ 0.5 A·g^**–1**^	**energy density @**5.6 mg (mW·h)	**power density @**5.6 mg (mW)	**capacitance/capacity and retention**(%)	**refs**
symmetric device, hierarchical porous carbon nanoparticles	0.13	4.68	55 F·g^–1^ @ 0.5 A·g^–1^ and 86.7% @ 5000 cycles	^75^
symmetric device, Schiff nitrogen-rich porous carbon nanosheets	0.70	22.91	284 F·g^–1^ @ 0.5 A·g^–1^ and 93.8% @ 10,000 cycles	^76^
symmetric device, polymer-supported nitrogen/oxygen-codoped porous carbons	0.18	6.48	76.1 F·g^–1^ @ 0.5 A·g^–1^ and 97.7% @ 7000 cycles	^72^
symmetric device, nitrogen/oxygen-codoped hierarchical porous carbon	1.01	21.38	180 F·g^–1^ @ 0.5 A·g^–1^ and 93.8% @ 10,000 cycles	^66^
asymmetric device, Schiff-based AC//Ni-MOF	0.18	1.34	75 C·g^–1^ @ 0.5 A·g^–1^ and 85.6% @ 5000 cycles	^77^
asymmetric device, Schiff-based Ni–OTTP AC//Ni OTTP	0.19	3.02	111.5 C·g^–1^ @ 0.5 A·g^–1^ and 75.4% @ 5000 cycles	^25^
asymmetric device, nickel-based Schiff AC//C3	0.24–0.11	4.2–12.2	98.3 C·g^–1^ @ 0.5 A·g^–1^ and 79.8% @ 10,000 cycles	in this work

a AC: activated carbon, MOF: metal organic framework, and OTTP: terephthalaldehyde.

For real-time applications, two asymmetric AC//C3 supercapacitor coin cells were connected in series, producing a combined voltage of ∼2.91 V, as shown in Figure 11A. This voltage was sufficient to power a 5 mm diameter red LED (2 V, 20 mA), as illustrated in Figure 11B. The light intensity of the LED was monitored over time and sustained up to ∼3.20 min, as displayed in Figure 11C–F. A schematic diagram, Scheme 2, demonstrated the overall electrochemical setup and the vital components used to fabricate the electrode (C3) and asymmetric device.

**Figure 11:**
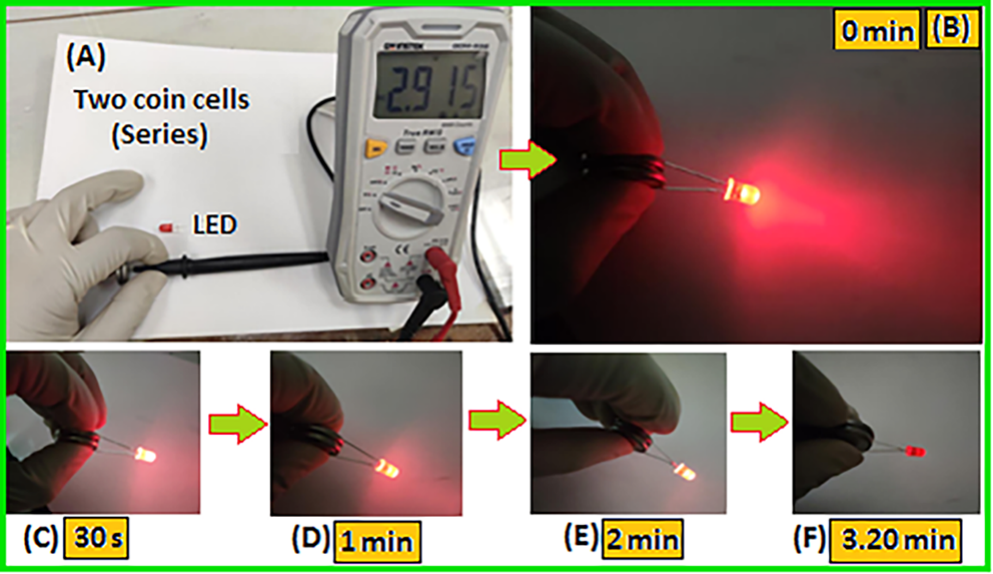
(A) Total voltage across two asymmetric coin cells connected in a series combination. (B) At the initial time (0 min), a red color LED was lit up by two coin cells. (C–F) The variation of light intensity was recorded at different time intervals (30 s to 3.20 min).

**Scheme 2:**
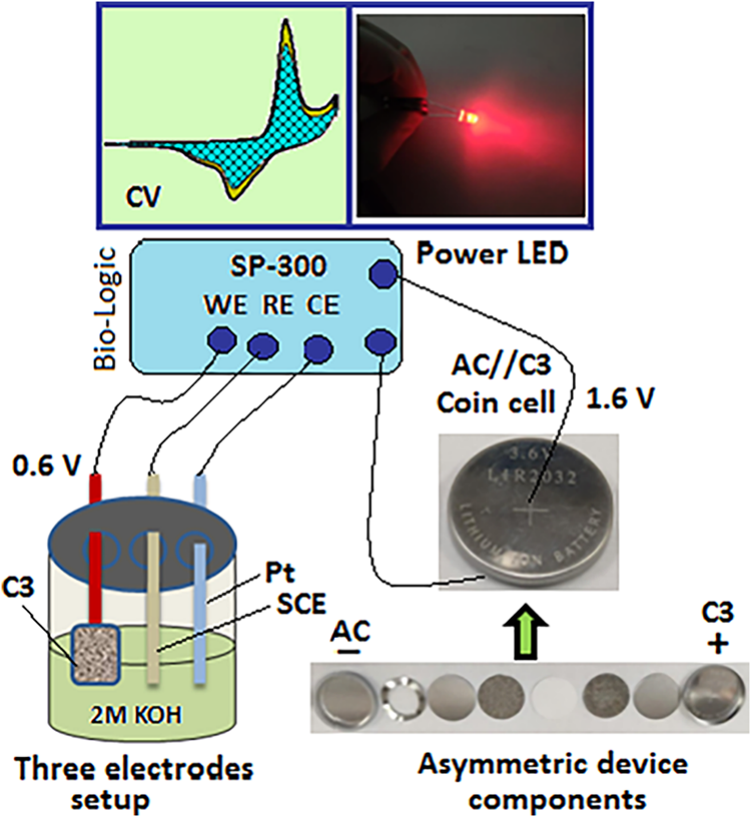
Schematic Diagram for Electrochemical Testing (Bio-Logic) by Using a Three-Electrode Setup under the 2M KOH Electrolyte; the Optimized Material (C3) was Fabricated into an Asymmetric Coin Cell AC//C3 to Power a Red LED

The electrochemical impedance spectra (EIS) of the C3 electrode and AC//C3 device were recorded over a frequency range from 200 kHz to 100 mHz, as shown in Figure 12A. The impedance spectra of the C3 electrode (in a three-electrode setup) and the AC//C3 device (in a two-electrode coin cell) were fitted (red lines) to an equivalent circuit model (Figure 12B,C, respectively) for the entire frequency range. The Nyquist plot for the C3 electrode was fitted using the equivalent circuit *R*_o_ – (*R*_ct_||*Q*_1_) – (*Q*_2_ + *W*). In this model, *R*_o_ corresponds to the intrinsic solution and contact resistance. The parallel *R*_ct_||*Q*_1_ branch represents the charge-transfer process coupled with nonideal double-layer capacitance at the electrode/electrolyte interface. The inclusion of the constant phase element (*Q*_1_) accounts for the surface heterogeneity and porosity of the Ni-foam-supported electrode. Surface roughness, heterogeneity, and the complex electrochemical process lead to deviation from ideal capacitor behavior. At lower frequencies, the combination of *Q*_2_ and Warburg impedance (W) reflects the pseudocapacitive response and diffusion of OH^–^ ions through the porous electrode structure. The near-vertical tail at low frequencies confirms capacitive diffusion control, while the small semicircle at high frequencies indicates efficient charge transfer and low resistance. The satisfactory fit with this model confirms that the electrochemical process involves a single interfacial charge-transfer reaction, followed by diffusion-controlled redox activity inside the C3 electrode.

**Figure 12:**
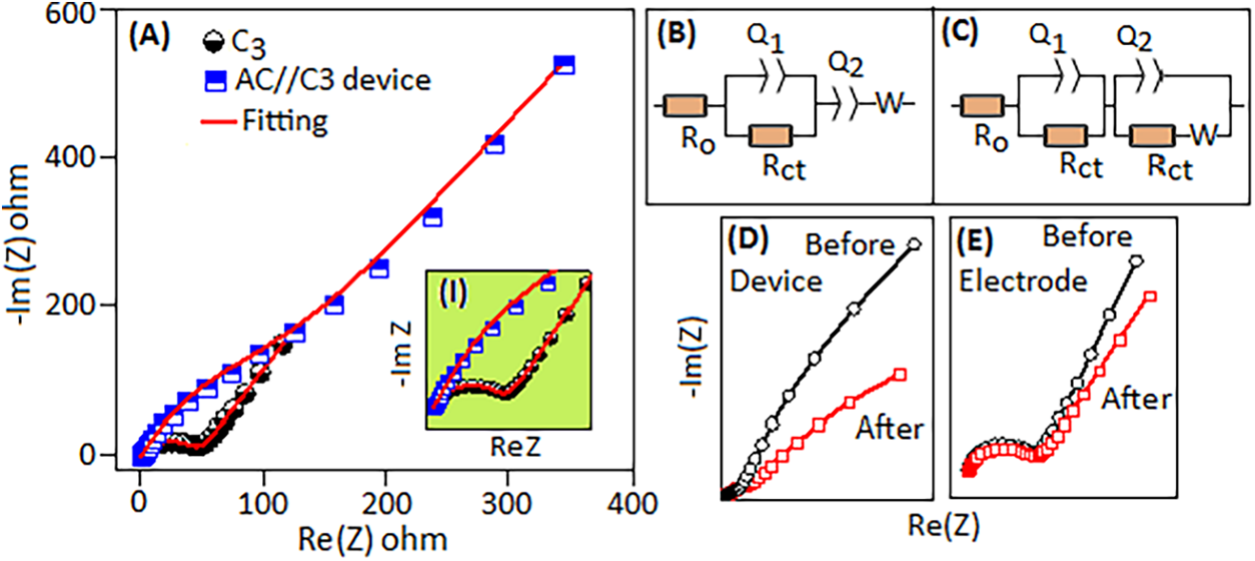
(A) Nyquist plot (Re *Z* vs −Im *Z*) of the C3 electrode and the AC//C3 device. The expanded view of the spectra is shown in the inset (I). The plots fitted (red line) according to the equivalent circuit model for (B) the C3 electrode and (C) the AC//C3 device. The EIS plot for the (D) device and the (E) electrode before and after 10,000 cycles.

For the C3//AC device, the curve fitted better with two parallel components, *R*_ct_||*Q*_1_ and *R*_ct_||*Q*_2_, that correspond to cathode–electrolyte interface (C3) and anode–electrolyte interface (activated carbon, AC) contributions, respectively, in the model. The nature of the Q_2_ element in the C3//AC device is the electrically double-layer capacitance (EDLC)-type non-Faradaic interaction between the AC (anode) electrode and electrolyte interface. In the asymmetric device, each electrode (positive and negative) has distinct kinetics and porosity, contributing to its relaxation time constant (τ = RC). The EIS response of the C3//AC device reflects both electrodes (cathode and anode) combined making the broader and smear semicircular arc compared to single prominent arc for C3 electrode, as shown in the expanded view inset (I) of Figure 12A. The fast (high-frequency) *R*_ct_||*Q*_2_ branch corresponds to electrolyte/interfacial contact resistance and fast double-layer charging, while the second (intermediate frequency) *R*_ct_||*Q*_1_ branch represents charge-transfer resistance and slower ion diffusion/pseudocapacitive storage in the positive electrode (C3). As the asymmetric device consists of two different electrodes with different kinetics, a two-branch equivalent circuit is essential to separately model each contribution. The device shows a higher impedance and a more vertical shift toward the low-frequency region, and it reflects the combined (both C3 and AC electrodes) diffusion and capacitive contributions in the Warburg diffusion element (W). The EIS spectra of the device, Figure 12D, and the C3 electrode, Figure 12E, were recorded before and after ≥10,000 charge–discharge cycles to evaluate the stability of the material on the Ni-foam substrate. The Nyquist plots reveal a clear increase in the diameter of the semicircle in the high-to-mid frequency region after cycling, indicating an increase in charge-transfer resistance. Additionally, the low-frequency region becomes more flattened toward the *x*-axis, implying higher diffusion resistance and reduced capacitive behavior due to pore blockage or active material detachment under prolonged cycling. The extracted fitting parameters from two equivalent models are listed in Table 3.

**Table 3:** Fitting Parameters Extracted from the Equivalent Circuit Model for the C3 Electrode and the Asymmetric AC//C3 Device over the Frequency Range from 200 kHz to 100 mHz

system	*R*_0_ (Ω)	*Q*_1_ (mF)	α_1_	*R*_ct_ (Ω)	*Q*_2_ (mF)	α_2_	*R*_ct_ (Ω)	*W* (Ω·s^–1/2^)	χ^2^
C3 electrode	1.85	6.75	0.88	40.4	1.67	0.83		40.6	2.12 × 10^–4^
AC//C3 device	3.12	50.9	0.82	13.0	2.9	0.90	41	31.2	1.31 × 10^–4^

## Conclusion

In summary, we present an in situ synthesis strategy for stabilizing three nickel­(II)-based Schiff base complexes derived from 2-hydroxybenzaldehyde with 2-bromo-4-chloroaniline (C1), 2-bromo-4-methylaniline (C2), and 2-iodo-4-nitroaniline (C3) without isolating the ligands. The structural homogeneity of the synthesized materials was characterized using standard spectroscopic techniques, with the solid-state structures of C1 and C2 determined through single-crystal X-ray diffraction analysis. Amid the escalating demand for portable electrochemical energy storage devices, the imperative lies in developing solutions that offer high energy densities, extended cycling durability, and cost-effectiveness. In this context, the C1, C2, and C3 complexes were fabricated as electrode materials for supercapacitors and evaluated for performance in a 2 M KOH aqueous electrolyte. The electrodes exhibited pseudocapacitive behavior, as evidenced by the redox-active peaks in cyclic voltammetry. Among these, the Schiff base-derived complex from 2-iodo-4-nitroaniline (C3) showed improved electrochemical activity and high dielectric polarizability. The C3 electrode delivered a maximum specific capacitance value of approximately 330 F·g^–1^ at a current density of 1 A·g^–1^, maintaining a reasonable long-term cycling stability of about ∼92.5% at 5 A·g^–1^ after 10,000 charge–discharge cycles. To materialize a practical energy storage prototype, an asymmetric coin cell (AC//C3) was devised, utilizing C3 as the positive (cathode) electrode and activated carbon as the negative (anode) electrode. This asymmetric cell operated at a working potential of 1.6 V and yielded a specific capacity of around 98.3 C·g^–1^ (∼61.4 F·g^–1^ or ∼27.3 mAh·g^–1^) at 0.5 A·g^–1^. The device shows a stable impedance response and a sustained Coulombic efficiency of ∼79.8% after 10,000 cycles, supporting a reversible Faradaic process without structural degradation. Notably, it exhibited an energy density of about 21.8 Wh·kg^–1^ with a power density of approximately 378.3 W·kg^–1^ at 0.5 A·g^–1^, reaching a peak power density of around 1089 W·kg^–1^ at 4.0 A·g^–1^. The electrochemical performance of the C3 electrode is consistent with other Ni-based asymmetric systems; however, the distinguishing contribution of this work is the integrated correlation among ligand design, dielectric/optical characteristics, and charge-storage behavior. This framework provides a rational basis for designing future molecularly tuned nickel­(II) complexes for supercapacitor applications. To demonstrate the real-time applicability of the device, two asymmetric (AC//C3) coin cells were connected in series to power a red LED. Two fully charged cells generated a voltage of approximately 2.91 V, ample to illuminate a red LED. Over successive intervals of 30 s, 1 min, 2 min, 3 min, and 20 s, the LED intensity gradually diminished, depicting the energy discharge characteristics of the 2-iodo-4-nitroaniline-stabilized nickel­(II)-based Schiff base complex as a promising electrode material for real-time energy storage applications.

## Supplementary Material







## Data Availability

Crystal information data: CCDC nos.: **2429322** and **2450772** revealed the crystallographic data for C1 and C2 and can be accessed from the Cambridge Crystallographic Data Centre at http://www.ccdc.cam.ac.uk/data_request/cif. Other datasets will be available on reasonable requests to the corresponding author.

## Notes

Spectra, electrochemical data, setups, and methodologies (PDF)
C1-ciffile (CIF)
C2-ciffile (CIF)
