# Supplementary material for: Nickel(II) Complexes Derived from Schiff Base Ligands Designed as Electrode Materials in Asymmetric Supercapacitor Coin Cells for Enhanced Energy Storage Performance
Source: Langmuir. 2026 Jan 12;42(3):2511–28. doi: 10.1021/acs.langmuir.5c04823 (PMC12856898; doi:10.1021/acs.langmuir.5c04823)
Supplement: Supplementary file 1 [file la5c04823_si_001.pdf]

Supporting Information

For

Nickel(II) Complexes Derived from Schiff Base Ligands Designed as Electrode Materials in Asymmetric Supercapacitor Coin Cells for Enhanced Energy Storage Performance

Ibrahim Waziri[a**], Tunde L. Yusuf[b], Alfred J. Muller[a], Charity N. Mbileni Morema[c], Kaushik Mallick[a], and Sarit K. Ghosh[a,c*]

[a]Department of Chemical Sciences, University of Johannesburg, P.O. Box: 524, Auckland Park 2006, South Africa.

[b]Department of Chemistry, Faculty of Natural and Agricultural Sciences, University of Pretoria, Private Bag X20, Hatfield 0028, Pretoria, South Africa

[c]NM Envirotech Solutions, Midlands Estate, Centurion 1692, South Africa.

[*]Corresponding author:triumph2236@gmail.com (I. Waziri) and saritghosh@gmail.com (S. Ghosh)

Table of contents

Figure S1: ^{1}H NMR spectrum of complex **C1** recorded at room temperature (500 MHz, DMSO-d_6), showing the signals of the proton atoms in the compound and their chemical environment.

Figure S2: ^{13}C NMR spectrum of complex **C1** recorded at room temperature (125 MHz, DMSO-d_6), showing the signals of the carbon atoms in the compound and their chemical environment

Figure S3: FTIR spectrum of complex **C1** recorded at room temperature in the solid-state using the ATR technique, showing the relevant stretching vibration bands and their frequencies.

Figure S4: UV–Vis electronic absorption spectrum of complex **C1** (solution state) obtained at room temperature using 10^{-3} M sample solution in DMSO, showing $\pi\rightarrow\pi^*$ (250 nm), $n\rightarrow\pi^*$ (300 nm), and d–d (435 nm) transitions. The moderately low-energy d–d band indicates a square-planar Ni(II) geometry with a medium ligand field and partial LMCT character.

Figure S5: High-resolution mass spectrum (HRMS) of complex **C1** recorded at room temperature.

Figure S6: ^{1}H NMR spectrum of complex **C2** recorded at room temperature (500 MHz, DMSO-d_6), showing the signals of the proton atoms in the compound and their chemical environment.

Figure S7: ^{13}C NMR spectrum of complex **C2** recorded at room temperature (125 MHz, DMSO-d_6), showing the signals of the carbon atoms in the compound and their chemical environment.

Figure S8: FTIR spectrum of complex **C2** recorded at room temperature in the solid-state using the ATR technique, showing the relevant stretching vibration bands and their frequencies

Figure S9: UV–Vis electronic absorption spectrum of **C2** (solution state) obtained at room temperature using 10^{-3} M sample solution in DMSO, showing $\pi\rightarrow\pi^*$ (274 nm), $n\rightarrow\pi^*$ (342 nm), and d–d (442 nm) transitions. The slightly blue-shifted d–d band and weaker CT features reflect a larger optical gap and weaker LMCT interaction, consistent with its lower electrochemical performance.

Figure S10: High-resolution mass spectrum (HRMS) of complex **C2** recorded at room temperature.

Figure S11: ^{1}H NMR spectrum of complex **C3** recorded at room temperature (500 MHz, DMSO-d_6), showing the signals of the proton atoms in the compound and their chemical environment.

Figure S12: ^{13}C NMR spectrum of complex **C3** recorded at room temperature (125MHz, DMSO-d_6), showing the signals of the carbon atoms in the compound and their chemical environment.

Figure S13: FTIR spectrum of complex **C3** recorded at room temperature in the solid-state using the ATR technique, showing the relevant stretching vibration bands and their frequencies.

Figure S14: UV–Vis electronic absorption spectrum of **C3** (solution state) obtained at room temperature using 10^{-3}M sample solution in DMSO, showing $\pi\rightarrow\pi^*$ (232 nm), $n\rightarrow\pi^*$ (290 nm), and d–d (530 nm) transitions. The pronounced red-shift and intensity of the low-energy band signify stronger LMCT/MLCT character and a reduced optical gap, correlating with the highest specific capacitance among the three complexes.

Figure S15: High-resolution mass spectrum (HRMS) of complex **C3** recorded at room temperature.

Table S1: Summary of UV–Vis electronic transitions of the complexes (**C1–C3**), highlighting the absorption band, energy and transitions.

Table S2: Crystal system data, X-ray data collection, and structure refinement details for complexes **C1** and **C2**

Figure S16: (a) XPS survey spectra of C3 material as pristine and post-cycle (cyclic voltammetry). Both spectra confirm the presence of Ni, C, N, O and I elements in the system. (b) High resolution Ni 2p peak behavior of pristine and post-cycle conditions

Figure S17: CV curves of the device obtained at different potential windows at 50 $mV.s^{-1}$ scan rate.

Figure S18: Dismantled images of the coin cell after 10000 cycles. No electrolyte leakage, salt crystallization or abnormal swelling was observed during the test.

Figure S19: Worked Example for two asymmetric devices

Spectra

C1

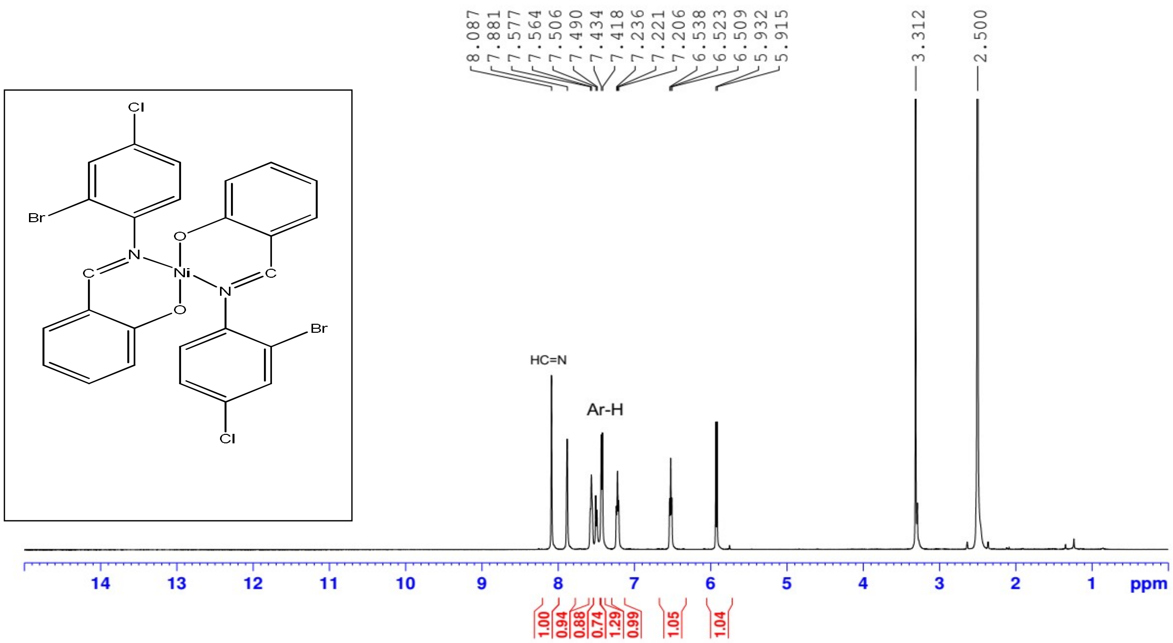

Figure S1: ^{1}H NMR spectrum of complex **C1** recorded at room temperature (500 MHz, DMSO-d_6), showing the signals of the proton atoms in the compound and their chemical environment.

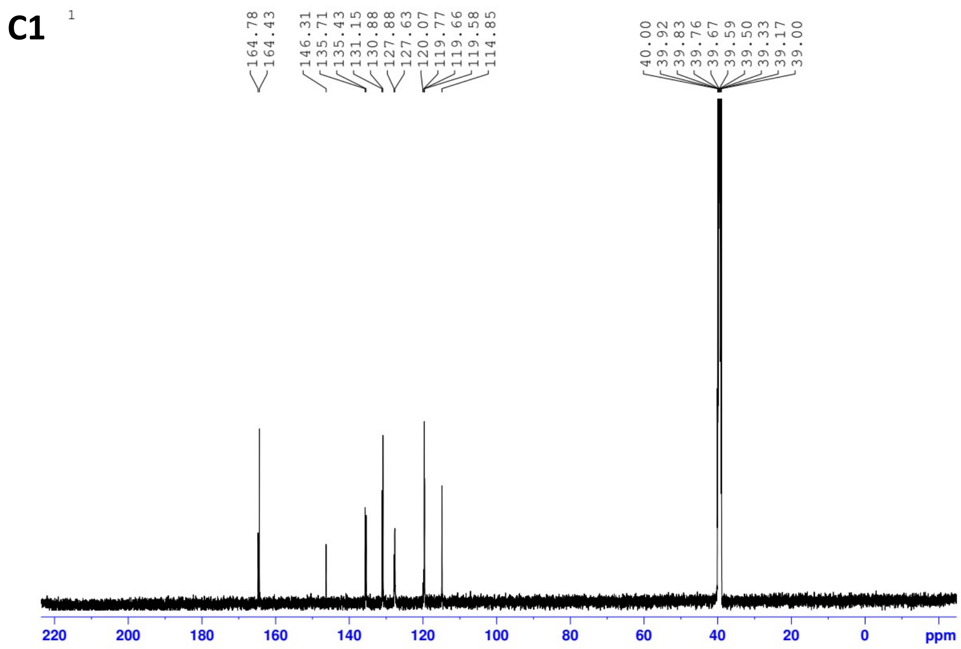

Figure S2: ^{13}C NMR spectrum of complex **C1** recorded at room temperature (125 MHz, DMSO-d_6), showing the signals of the carbon atoms in the compound and their chemical environment.

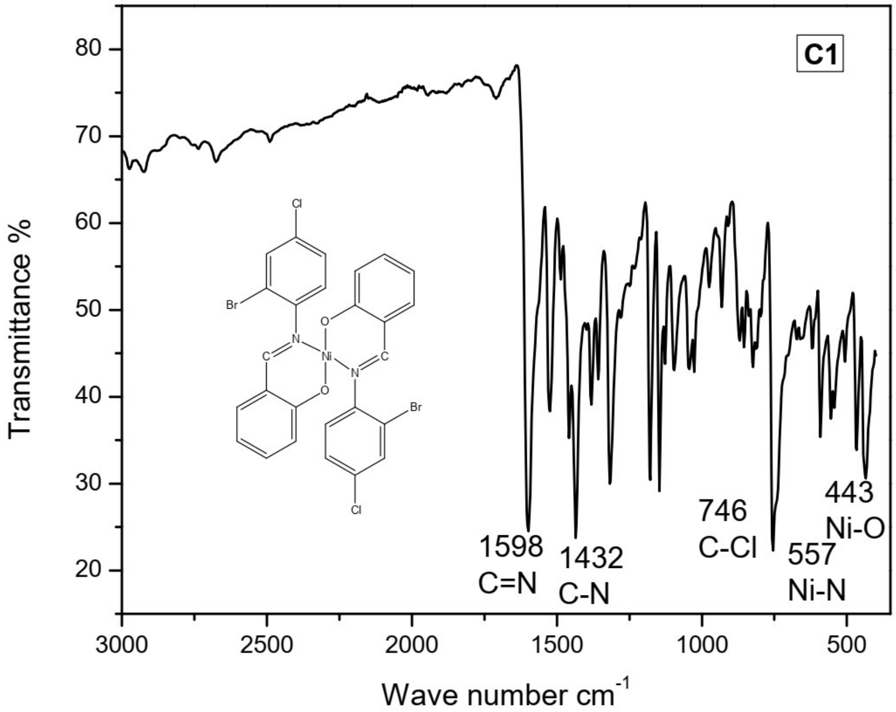

Figure S3: FTIR spectrum of complex **C1** recorded at room temperature in the solid-state using the ATR technique, showing the relevant stretching vibration bands and their frequencies.

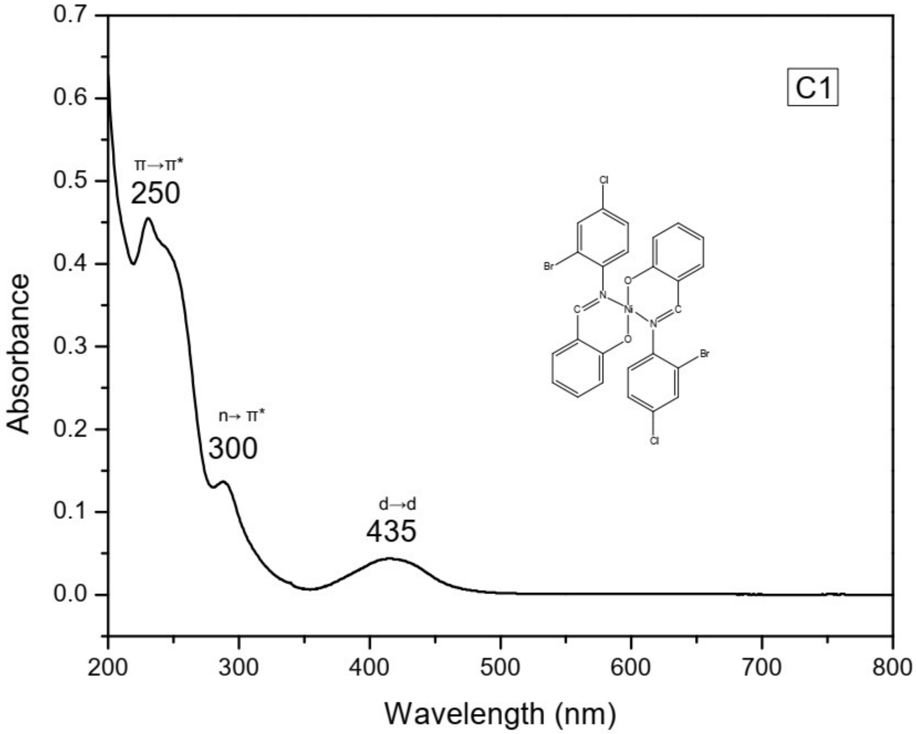

Figure S4: UV–Vis electronic absorption spectrum of complex **C1** (solution state) obtained at room temperature using 10^{-3} M sample solution in DMSO, showing π→π* (250 nm), n→π* (300 nm), and d–d (435 nm) transitions. The moderately low-energy d–d band indicates a square-planar Ni(II) geometry with a medium ligand field and partial LMCT character.

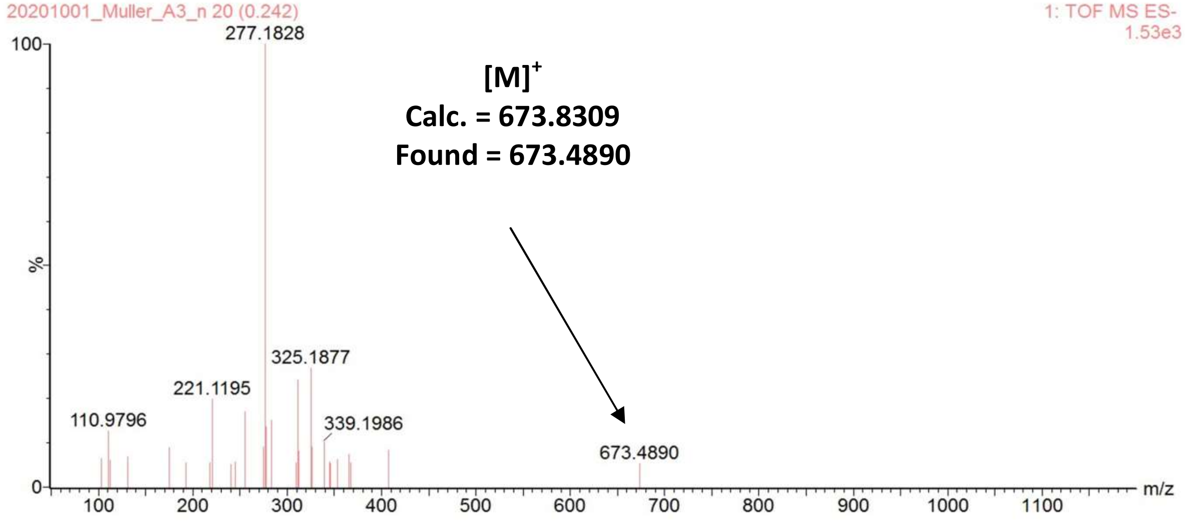

Figure S5: High-resolution mass spectrum (HRMS) of complex **C1** recorded at room temperature.

C2

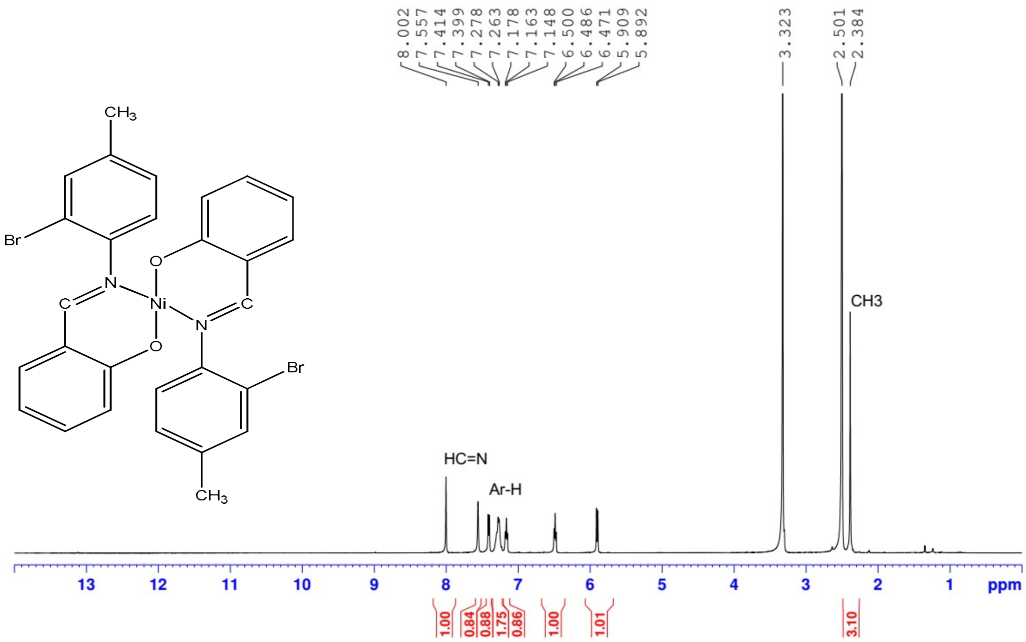

Figure S6: ^{1}H NMR spectrum of complex **C2** recorded at room temperature (500 MHz, DMSO-d_6), showing the signals of the proton atoms in the compound and their chemical environment.

C2

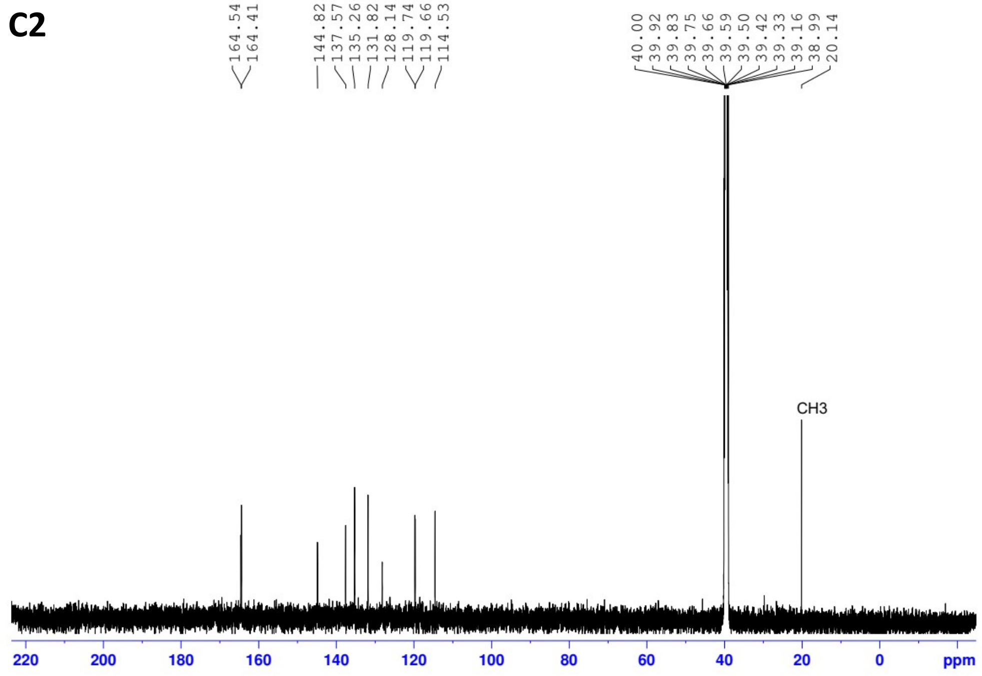

Figure S7: ^{13}C NMR spectrum of complex **C2** recorded at room temperature (125 MHz, DMSO-d_6), showing the signals of the carbon atoms in the compound and their chemical environment.

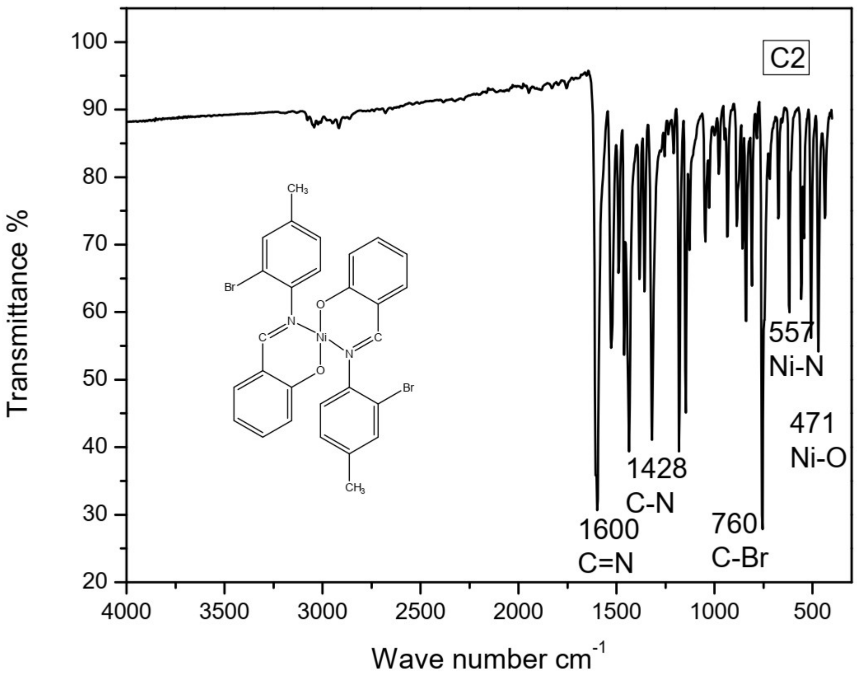

Figure S8: FTIR spectrum of complex **C2** recorded at room temperature in the solid-state using the ATR technique, showing the relevant stretching vibration bands and their frequencies.

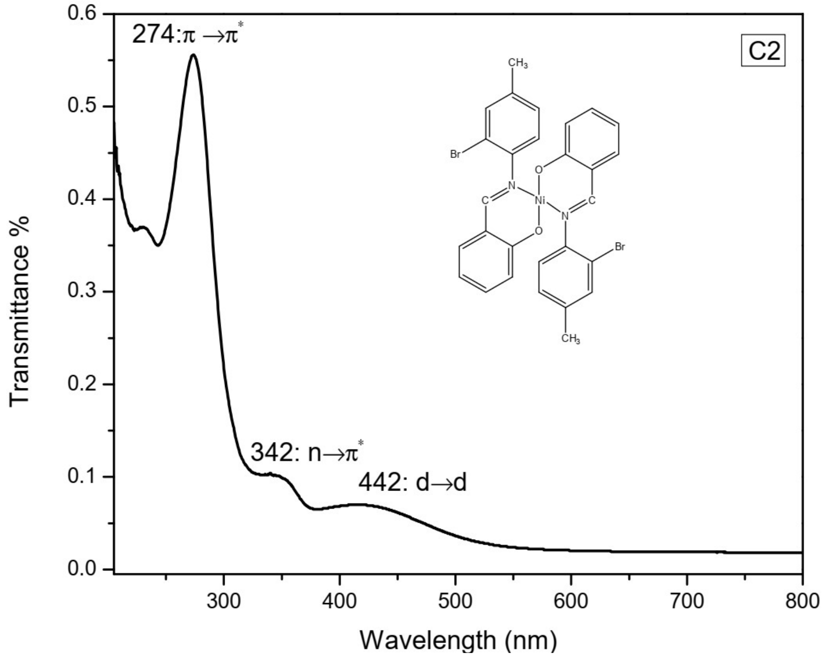

Figure S9: UV–Vis electronic absorption spectrum of **C2** (solution state) obtained at room temperature using 10^{-3} M sample solution in DMSO, showing π→π* (274 nm), n→π* (342 nm), and d–d (442 nm) transitions. The slightly blue-shifted d–d band and weaker CT features reflect a larger optical gap and weaker LMCT interaction, consistent with its lower electrochemical performance.

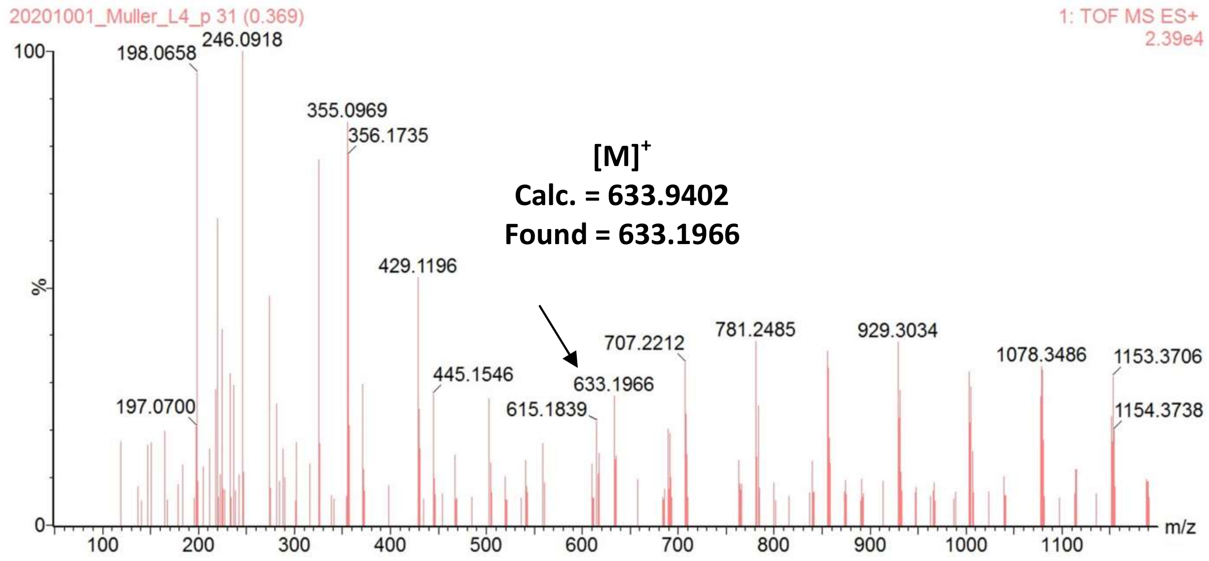

Figure S10: High-resolution mass spectrum (HRMS) of complex **C2** recorded at room temperature.

C3

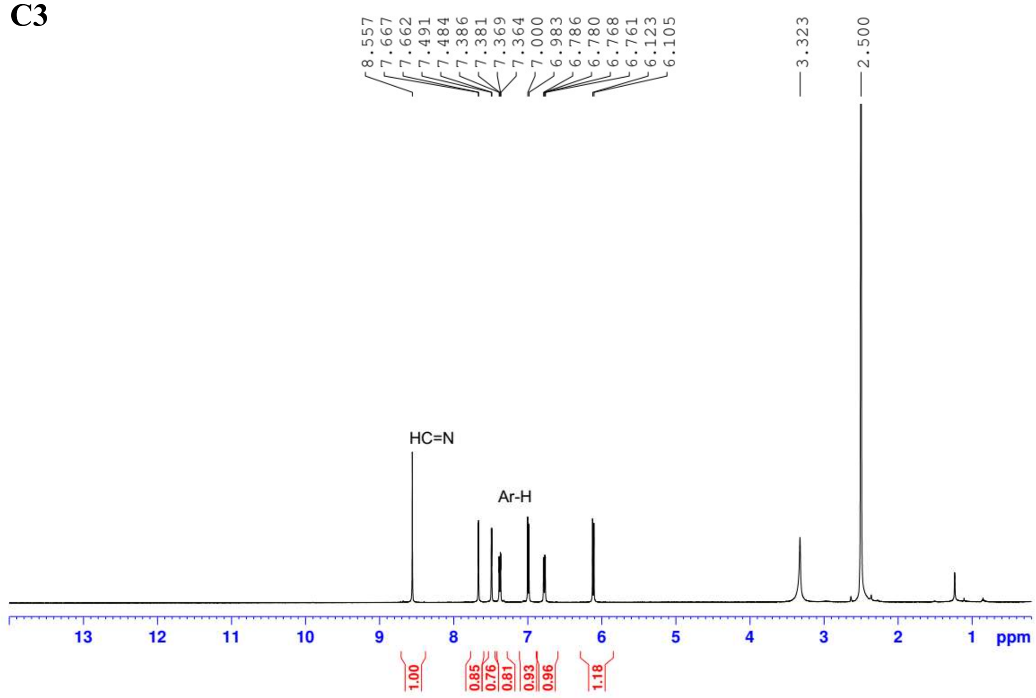

Figure S11: ^{1}H NMR spectrum of complex **C3** recorded at room temperature (500 MHz, DMSO-d_6), showing the signals of the proton atoms in the compound and their chemical environment.

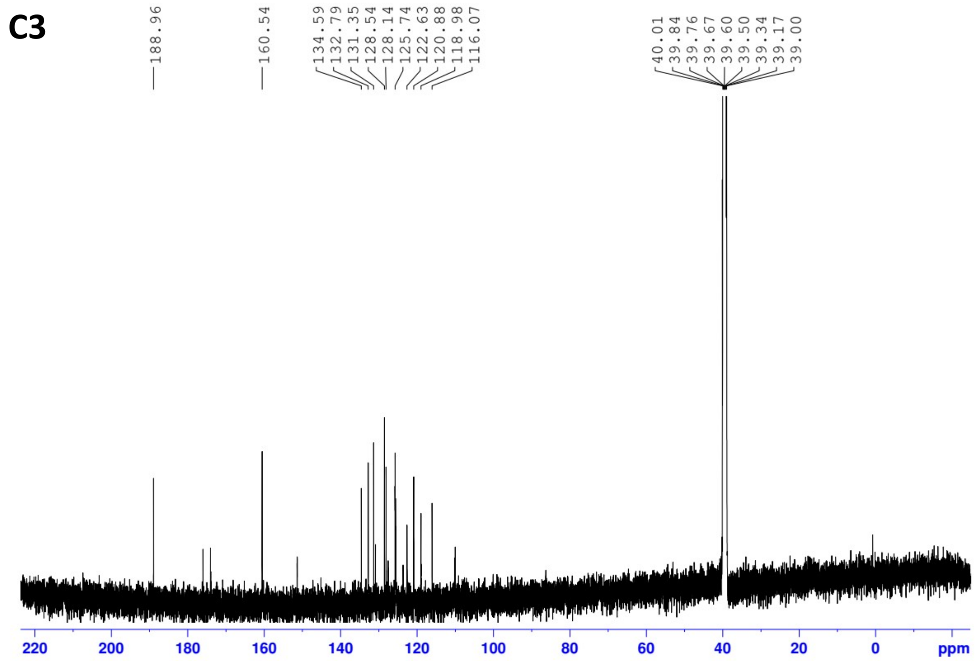

Figure S12: ^{13}C NMR spectrum of complex **C3** recorded at room temperature (125MHz, DMSO-d_6), showing the signals of the carbon atoms in the compound and their chemical environment.

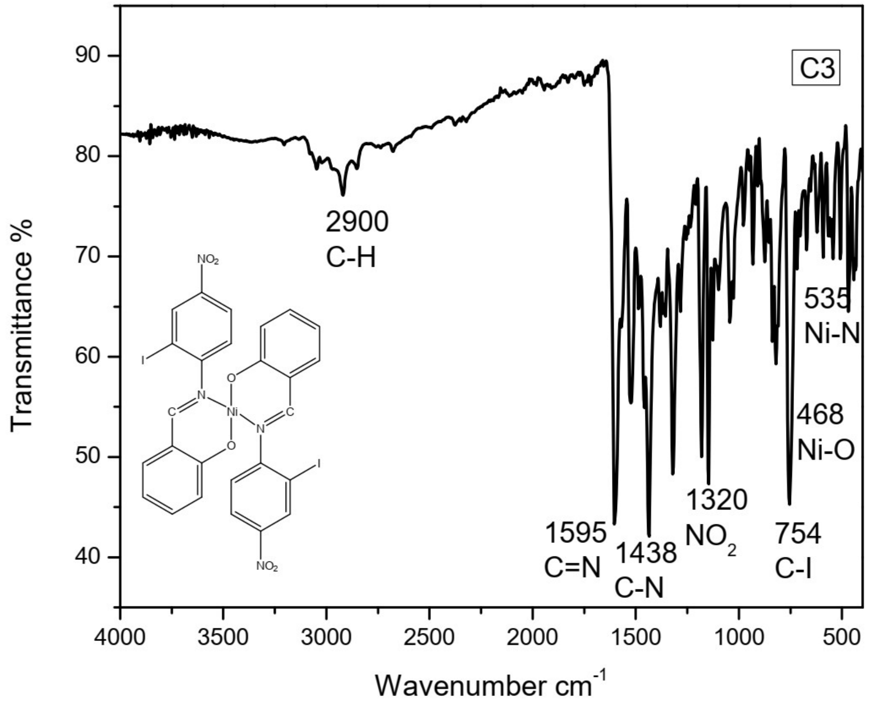

Figure S13: FTIR spectrum of complex **C3** recorded at room temperature in the solid-state using the ATR technique, showing the relevant stretching vibration bands and their frequencies.

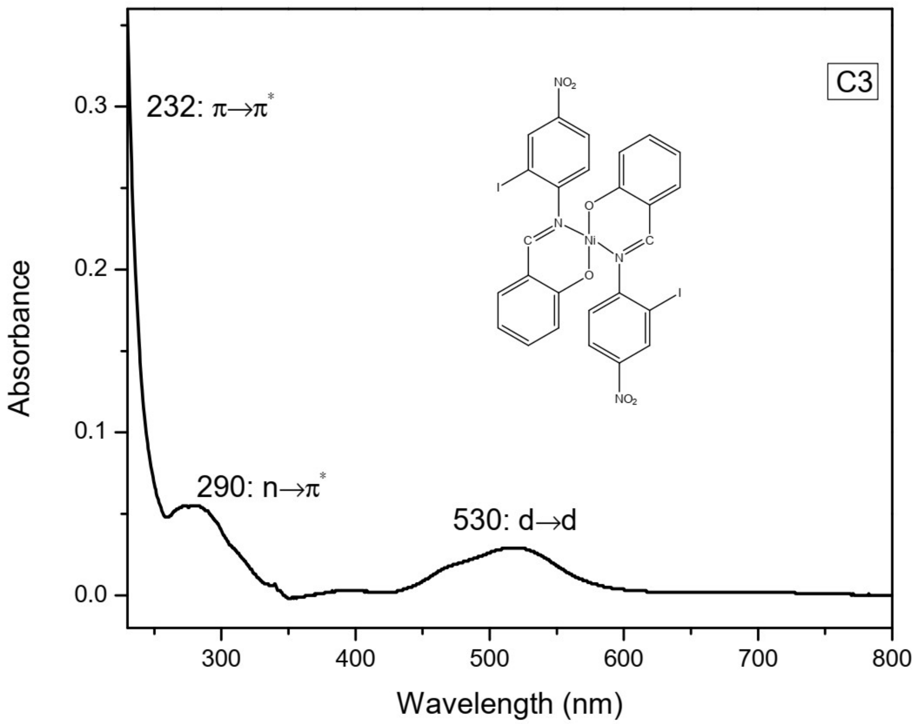

Figure S14: UV–Vis electronic absorption spectrum of **C3** (solution state) obtained at room temperature using 10^{-3}M sample solution in DMSO, showing π→π* (232 nm), n→π* (290 nm), and d–d (530 nm) transitions. The pronounced red-shift and intensity of the low-energy band signify stronger LMCT/MLCT character and a reduced optical gap, correlating with the highest specific capacitance among the three complexes.

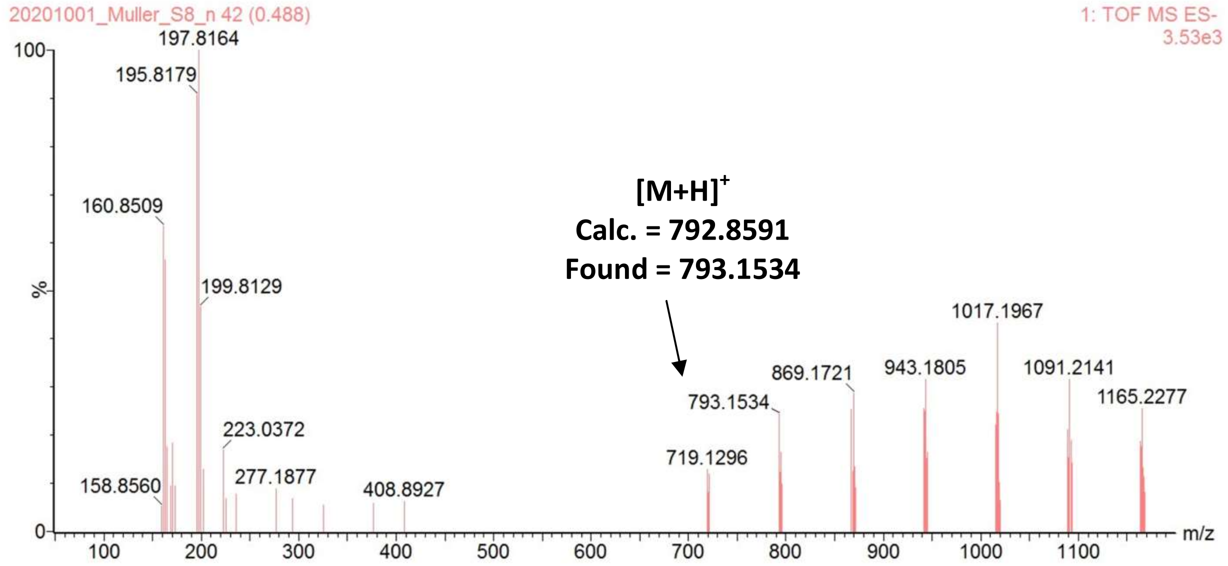

Figure S15: High-resolution mass spectrum (HRMS) of complex **C3** recorded at room temperature.

Table S1: Summary of UV–Vis electronic transitions of the complexes (**C1–C3**), highlighting the absorption band, energy and transitions.

Complexes	Transitions	λ_{max} (nm)	Energy (eV)	Remarks
**C1**	π→π*	250	4.96	Aromatic ring transition
	n→π*	300	4.13	Azomethine (C=N) lone-pair excitation
	d–d	435	2.85	Ni(II) square-planar; moderate LMCT contribution
**C2**	π→π*	274	4.53	Aromatic ring transition
	n→π*	342	3.63	Azomethine (C=N) lone-pair excitation
	d–d	442	2.81	Ni(II) square-planar; weaker ligand field (methyl substituent)
**C3**	π→π*	232	5.35	Aromatic ring transition (blue-shifted)
	n→π*	290	4.28	Azomethine (C=N) lone-pair excitation
	d–d	530	2.34	Ni(II) square-planar; strong LMCT/MLCT, reduced optical gap

Note: UV–Vis electronic transitions for the complexes **C1–C3**. The lower-energy d–d/LMCT band in C3 (530 nm, 2.34 eV) indicates enhanced charge-transfer character and a smaller optical gap, consistent with its superior performance as a Supercapacitor electrode material (**C3 > C1 > C2**).

Table S2: Crystal system data, X-ray data collection, and structure refinement details for complexes **C1** and **C2**

**Identification code**	**C1**	**C2**
Empirical formula	$C_{26}H_{16}Br_2Cl_2N_2NiO_2$	$C_{28}H_{22}Br_2N_2NiO_2$
Formula weight	677.834	637.00
Temperature/K	273.15	149.99(10)
Crystal system	monoclinic	monoclinic
Space group	C2/c	$P2_1/c$
a/Å	21.3488(6)	10.6411(4)
b/Å	6.7330(2)	12.1530(3)
c/Å	18.0467(5)	10.6481(5)
α/°	90	90
β/°	97.030(1)	115.175(5)
γ/°	90	90
Volume/$Å^3$	2574.56(13)	1246.23(9)
Z	8	2
$\rho_{calc}g/cm^3$	1.749	1.698
μ/mm^{-1}	4.092	4.014
F(000)	1337.7	636.0
Crystal size/mm^3	0.345 × 0.193 × 0.182	0.111 × 0.111 × 0.111
Radiation	Mo Kα (λ = 0.71073)	Mo Kα (λ = 0.71073)
2Θ range for data collection/°	3.84 to 55.78	5.394 to 58
Index ranges	-28 ≤ h ≤ 28, -8 ≤ k ≤ 8, -23 ≤ l ≤ 23	-13 ≤ h ≤ 13, -16 ≤ k ≤ 16, -13 ≤ l ≤ 14
Reflections collected	26620	12354

Independent reflections	3072 [R_{int} = 0.0541, R_{sigma} = 0.0318]	3041 [R_{int} = 0.0200, R_{sigma} = 0.0175]
Data/restraints/parameters	3072/0/160	3041/0/161
Goodness-of-fit on F^2	1.089	1.043
Final R indexes [I>=2σ (I)]	R_1 = 0.0270, wR_2 = 0.0726	R_1 = 0.0231, wR_2 = 0.0565
Final R indexes [all data]	R_1 = 0.0316, wR_2 = 0.0750	R_1 = 0.0282, wR_2 = 0.0581
Largest diff. peak/hole / e Å^{-3}	0.63/-0.61	0.45/-0.50

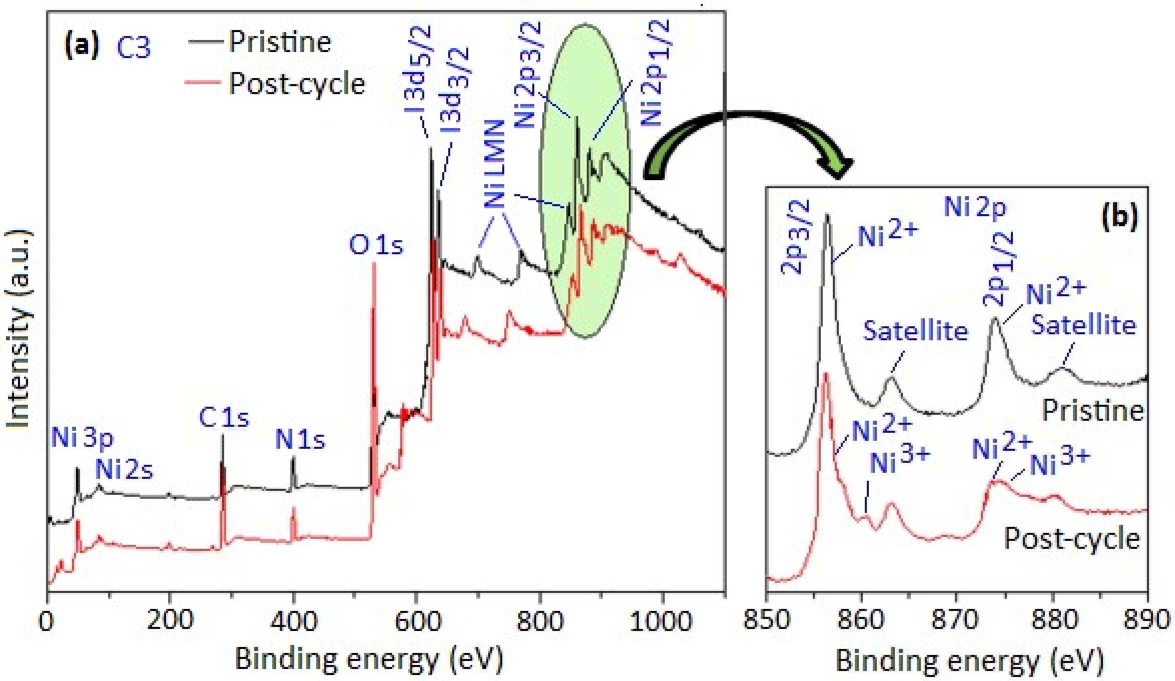

Figure S16: (a) XPS survey spectra of C3 material as pristine and post-cycle (cyclic voltammetry). Both spectra confirm the presence of Ni, C, N, O and I elements in the system. (b) High resolution Ni 2p peak behavior of pristine and post-cycle conditions.

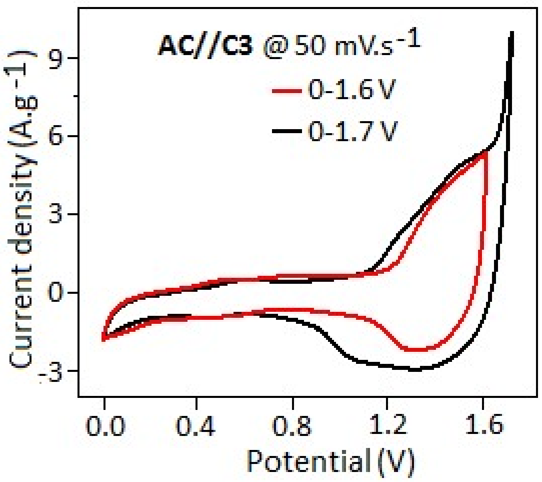

Figure S17: CV curves of the device obtained at different potential windows at 50 mV.s^{-1} scan rate.

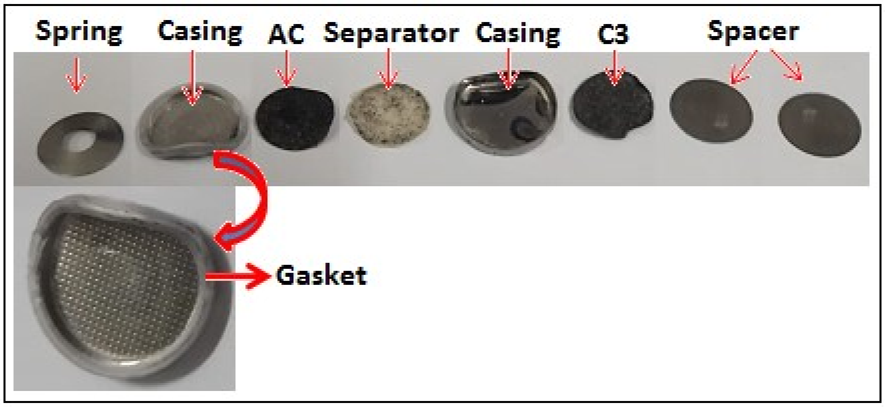

Figure S18: Dismantled images of the coin cell after 10000 cycles. No electrolyte leakage, salt crystallization or abnormal swelling was observed during the test.

Worked Example for two asymmetric devices

Recalculation of E and P values at same active mass (5.6 mg), same potential (1.6 V) and same current density (0.5 A.g^{-1})

1. Asymmetric device (AC//Ni OTTP) Schiff based Ni-OTTP system

The energy density (E) = 34.5 Wh.kg^{-1} and power density (P) = 613 W.kg^{-1} at 0.5 A.g^{-1} (given values).
ΔV = 1.6 V, Δt ~230 s (estimated from GCD curve at 0.5 A.g^{-1}).

Recalculation of E and P values using active mass (5.6 mg) and same discharge window (1.6 V).

For, 5.6 mg = 5.6 × 10^{-6} kg,

$E = (34.5/10^6) \times 5.6 = 0.000193$ Wh = **0.193 mWh**

$P = (E \times 3600/t) = 0.193 \times (3600/230) =$ **3.02 mW**.

2. Asymmetric device (AC//Ni-MOF) Schiff based system

Specific capacity (Q) = 75 C.g-1, (20.83 mAh.g^{-1}) at 0.5 A.g^{-1} (given values).

V = 1.6 V, $\Delta t \sim 500$ s (estimated from GCD curve at 0.5 A.g^{-1}).

The energy density (E) = 20.83 mAh.$g^{-1} \times$ 1.6 V = 33.33 Wh.kg^{-1}

For, 5.6 mg = 5.6×10^{-6} kg

$E = (33.3/10^6) \times 5.6 = 0.000186$ Wh = **0.186 mWh**

$P = 0.183 \times (3600/500) =$ **1.34 mW**.
